# Charge Trapping and Defect Dynamics as Origin of Memory Effects in Metal Halide Perovskite Memlumors

**DOI:** 10.1021/acs.jpclett.4c00985

**Published:** 2024-06-06

**Authors:** Alexandr Marunchenko, Jitendra Kumar, Alexander Kiligaridis, Shraddha M. Rao, Dmitry Tatarinov, Ivan Matchenya, Elizaveta Sapozhnikova, Ran Ji, Oscar Telschow, Julius Brunner, Alexei Yulin, Anatoly Pushkarev, Yana Vaynzof, Ivan G. Scheblykin

**Affiliations:** 1 Chemical Physics and NanoLund, Lund University, P.O. Box 124, 22100 Lund, Sweden; 2 School of Physics and Engineering, ITMO University, 49 Kronverksky, St. Petersburg 197101, Russian Federation; 3 Chair for Emerging Electronic Technologies, Technical University of Dresden, Nöthnitzer Straße 61, 01187 Dresden, Germany; 4 Leibniz-Institute for Solid State and Materials Research Dresden, Helmholtzstraße 20, 01069 Dresden, Germany

## Abstract

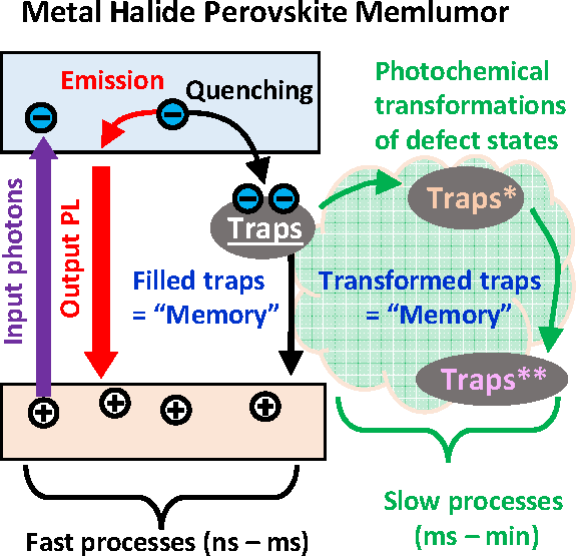

Large language models for artificial intelligence applications require energy-efficient computing. Neuromorphic photonics has the potential to reach significantly lower energy consumption in comparison with classical electronics. A recently proposed memlumor device uses photoluminescence output that carries information about its excitation history via the excited state dynamics of the material. Solution-processed metal halide perovskites can be used as efficient memlumors. We show that trapping of photogenerated charge carriers modulated by photoinduced dynamics of the trapping states themselves explains the memory response of perovskite memlumors on time scales from nanoseconds to minutes. The memlumor concept shifts the paradigm of the detrimental role of charge traps and their dynamics in metal halide perovskite semiconductors by enabling new applications based on these trap states. The appropriate control of defect dynamics in perovskites allows these materials to enter the field of energy-efficient photonic neuromorphic computing, which we illustrate by proposing several possible realizations of such systems.

Solution-processed metal halide perovskite (MHP) semiconductors have been intensively studied since 2009 when their potential as photovoltaic materials was rediscovered. After a decade of research, MHP solar cells have reached power conversion efficiencies of 26.2%.^1−9^ Moreover, bright photoluminescence (PL) of MHPs manifested by the high photoluminescence quantum yield (PLQY) allows them to enter the application fields of light-emitting diodes (LED) displays, imaging, and sensing.^10−15^ The advantage of this material for industrial applications lies in the simplicity of its solution-processing at low temperatures, which can be integrated with large-scale fabrication technologies such as slot-die, inkjet-printing and spray coating.^16,17^

A major drawback of solution-processable MHPs preventing their commercialization is the limited device stability.^18−20^ Metastability of device responses originates from the abundance of physical and chemical processes^21−28^ related to the dynamic ionic nature of MHPs allowing for restructuring and adaptation of the crystal structure in response to external stimuli like electrical current, voltage and light irradiation. However, these instabilities can play a positive role, allowing MHPs to enter the field of neuromorphic computing. For example, controlling ion dynamics makes it possible to realize electrical or optoelectrical memristors based on MHPs.^29−35^

Very recently, we proposed a novel concept that relies on the use of the intrinsic metastability of MHPs’ PL: a memlumor, a luminophore with memory.^36^ The concept is based on the use of the PL intensity as an output signal which “remembers” the history of the excitation light (input signal) due to photoinduced processes in the material. MHPs are known for their photosensitivity and therefore are highly suitable materials for the realization of the memlumor concept.^36^ We showed that the PLQY of a CsPbBr_3_ thin film is able to store the history of input excitation in wide range of time scales from ns to min and operates down to fJ energy scale for one switching operation.^36^

In this work, the memlumor properties of MHPs are studied theoretically and experimentally. Using the general framework of the Shockley–Read–Hall charge recombination model, we are able to explain the memlumor response of a CsPbBr_3_ polycrystalline thin film on time scales from nanoseconds to minutes. This allows us to match the general theoretical formalism of the so-called state vector ***X⃗*** that is used to describe neuromorphic responses with the Shockley–Read–Hall recombination model. We found that the state vector ***X⃗***, which determines the PLQY of the MHP memlumors, must contain variables describing dynamics of the charge trapping and recombination coefficients of the defect states. Finally, we demonstrate the potential of memlumor applications for photonic neuromorphic computing.

The concept of memlumor introduced in our recent work^36^ requires the formalism of the state vector ***X⃗*** (Figure 1a). The state vector ***X⃗*** in memlumors essentially represents the memory, which, generally, can be written, read, or erased. The state vector ***X⃗*** consists of a set of dynamic variables reflecting the physical or chemical changes induced by light input *I*(*t*) in the material. The dynamics of these variables is determined by the differential equation on the state vector d***X⃗***/d*t* = *F*(***X⃗***, *I*(*t*)). Because these variables impact the output photoluminescence PL(*t*), they should be included in the transfer function between input and output, which is the state-dependent photoluminescence quantum yield PLQY(***X⃗***, *I*(*t*)) (Figure 1a). As a result, the same material may give substantially different PL(*t*) under the same momentary light excitation conditions *I*(*t*), because of the state vector ***X⃗*** difference induced by variations in the previous excitation history. Within the presented formalism, the PLQY(***X⃗***, *I*(*t*)) function can be considered as the synaptic weight of memlumors^36^ which can be changed by light irradiation.

**Figure 1:**
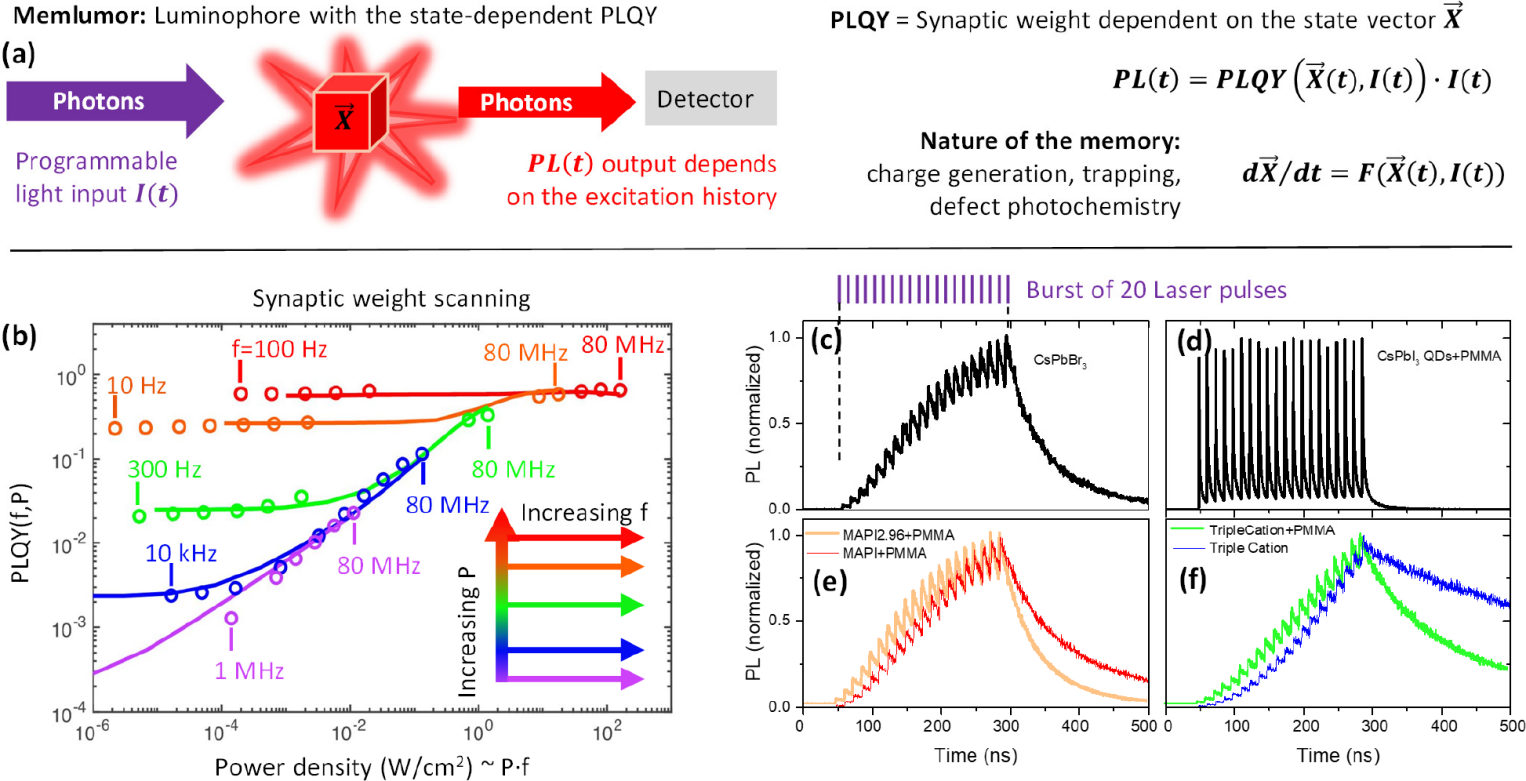
The concept of memlumor, synaptic weight, and short-term memory in metal halide perovskites. (a) The memlumor is a device that transforms the input of incoming high-energy photons into the output of lower-energy photons photoluminescence (PL). The concept of a memlumor requires the presence of the internal state of the system represented by the state vector ***X⃗***. This state vector ***X⃗*** defines the memlumor photoluminescence quantum yield (PLQY) which can be considered as the synaptic weight of the memlumor. The nature of the state vector may include many physical and chemical processes such as charge generation, trapping, or photochemical material transformations. (b) The PLQY of any luminescent material can be plotted in the parameter space of laser excitation pulse repetition rate (*f*) and pulse fluence (*P*). PLQY(*f*,*P*) is the pulse repetition rate and pulse fluence dependence of the memlumor synaptic weight. Here the pulse fluence varies from 0.14 nJ/cm^2^ (purple color) to 2000 nJ/cm^2^ (red color). This PLQY(*f*,*P*) plot shows data (circles) measured for CsPbBr_3_ polycrystalline thin film, while the solid lines correspond to the modeling (Supplementary Note 5). (c–f) The response of the PL under the pulse burst excitation is tracked for various types of metal halide perovskites. Here a pulse burst of 20 pulses with a pulse fluence of 17.6 nJ/cm^2^ is repeated each 62.5 μs. While the CsPbBr_3_ polycrystalline thin film shows pronounced modulation of pulse-to-pulse PL, the CsPbI_3_ QDs thin film covered by PMMA (d) lacks PL modulation. The composition of MHPs impacts the shape of the pulse-to-pulse memlumor response (e) as well as the additional passivation layers (f).

It is well-known that PL of many semiconductors grows nonlinearly upon increasing of the excitation power.^37−40^ It means that their PLQY is dependent on the excitation conditions. This fact alone means that, in principle, such materials have properties inherent to memlumors. Recently some of us demonstrated and applied to MHPs a comprehensive experiment designed to probe PLQY over a very broad space of excitation conditions realized by a pulsed laser source (Supplementary Note 2.1) .^41−43^ This method is called PLQY(*f*,*P*) mapping, where *P* is pulse fluence and *f* is the pulse repetition rate (Supplementary Notes 2.4 and 3). The map consists of a series of dependencies of PLQY on the time-averaged excitation power density (W/cm^2^, proportional to *f*·*P*) obtained for several selected fluences (*P*). In each curve *P* is fixed, but *f* is scanned over a very broad range. This presentation is very informative as discussed in detail elsewhere.^41^ In the context of memlumors, PLQY(*f*,*P*) shows the dependence of the synaptic weight at the periodic pulsed excitation condition on the pulse fluence and the period of pulsing.

PLQY(*f*,*P*) map for the CsPbBr_3_ thin film (Supplementary Note 1) investigated here is presented in Figure 1b. Lines of different colors are the results of modeling (see below). Each color represents the PLQY when excited at a fixed *P* value, while the repetition frequency is scanned from tens of Hz to 80 MHz. The laser pulse width is approximately 200 ps. The pulse fluence was set to 5 different values (*P*_1_, *P*_2_, ..., *P*_5_) with 1 order of magnitude difference between the sequential *P* values (Supplementary Notes 2.2, 2.4, and 3). The map shows that the PLQY can differ by several orders of magnitude depending on the excitation condition. For example, the same averaged excitation power density of 0.01 W/cm^2^, gives the same PLQY for the pulse energies *P*_1_ and *P*_2_, while it is ≈1.5 times higher for *P*_3_, ≈12 times higher for *P*_4_, and ≈30 times higher for *P*_5_. Note that the repetition rate for all of these points is scaled accordingly to keep the average power the same. So, a low repetition rate but a high pulse fluence can give a higher PLQY than a high repetition rate and a low pulse fluence. Another note is that for each pulse fluence, we observe the so-called single-pulse regime^41^ for low *f* where the PLQY is repetition frequency-independent (horizonal lines). However, increasing *f* toward 80 MHz generally modifies the PLQY which can increase by several orders of magnitude. This strong frequency-dependent response of PLQY(***X⃗***, *I*(*t*)) is a starting point for further considering a luminescent material as a potentially efficient memlumor.

While mapping the PLQY over the laser pulse fluence and repetition rate gives the steady-state values of PLQY under different excitation regimes, it is even more informative to resolve the history-dependent PL dynamics in time at the ns−μs time scale. For this, we apply an unusual excitation scheme in which PL is excited by pulse bursts. Figure 1c–f shows time-resolved PL responses of several MHP compositions to a burst of 20 pulses with 12.5 ns between each of them. The response was measured by a TCSPC setup with the repetition period of the pulse sequence equal to 62.5 μs (see Supplementary Note 2.3).^36^

The quasicontinuous growth of PL in response to the pulse burst that is observed for CsPbBr_3_ resembles the behavior of a leaky integrator. Here, the PL signal is enhanced by each next excitation pulse (being “integrated”) due to the memory effect while between the pulses the signal goes down (“leaks”) due to charge carrier recombination. The combination of these two effects makes the integrated PL response of the last pulse of the burst around 50 times higher than that to the first pulse of the burst, which can be referred to as a potentiation behavior.^44−47^

To examine whether this potentiation behavior is a general property of the MHP material class, we study several other MHP samples with different composition and morphology (Supplementary Note 1) (Figure 1d–f). In the case of methylammonium lead triiodide (MAPbI_3_) and triple cation (CsMAFA) thin films, we also observe a clear potentiation behavior (Figure 1e,f) that is, however, different for various compositions. Moreover, the response can be further modified by either defect engineering or interfacial modification. The former is illustrated for MAPbI_3_, where a vacancy-rich sample was prepared by lowering the stoichiometry to 2.96 ^42,48^ (Figure 1e). The impact of interfacial modification using poly(methyl methacrylate) (PMMA) as an example is illustrated for the CsMAFA case (Figure 1f). Importantly, the potentiation behavior is not ubiquitous to all perovskite samples. For example, for the same excitation conditions we did not observe the potentiation behavior in a film of CsPbI_3_ perovskite quantum dots (Figure 1d). This matches well the fact that the PLQY of materials where excited states are excitons is constant over the broad range of power densities (as in the case of semiconductor quantum dots). Thus, the PLQY of excitonic materials cannot “remember” excitation history because PLQY is not excitation intensity-dependent. As for bulk MHPs, they all possess excitation intensity dependent PLQY in the range of pulse fluences used in the experiments (Supplementary Table 1), and thus, their PLQY can be modulated by light. Note that this power dependence does not contradict the presence of a certain fraction of excitons in the dynamic equilibrium with free charge carriers in bulk MHPs. This fraction for the studied metal halide perovskites at the condition of room temperature and low excitation fluences used is minor.^49,50^

In order to demonstrate the memory effect in an even more direct experiment, we measured the PL response in the so-called Write–Read excitation scheme, where the memory created by a burst of laser pulses (as discussed before) is “read” after a certain delay by evaluation of the PL response to a single-pulse excitation. Figure 2 demonstrates the PL response of the CsPbBr_3_ film to this excitation scheme for 40 Write pulses and a Read pulse arriving with a 0.55 μs delay after the end of the Write burst. The PL response generated by the Read pulse is approximately 3 times larger than the response to the first pulse of the Write burst. So, the Write burst enhances the single pulse response (i.e., PLQY at these conditions) by a factor of 3 when measured 0.55 μs after the end of the Write burst. As will be shown below, the origin of this memory is charge trapping, the process that “stores” information about the light excitation history in the form of trapped, e.g., electrons in the defect states.

**Figure 2:**
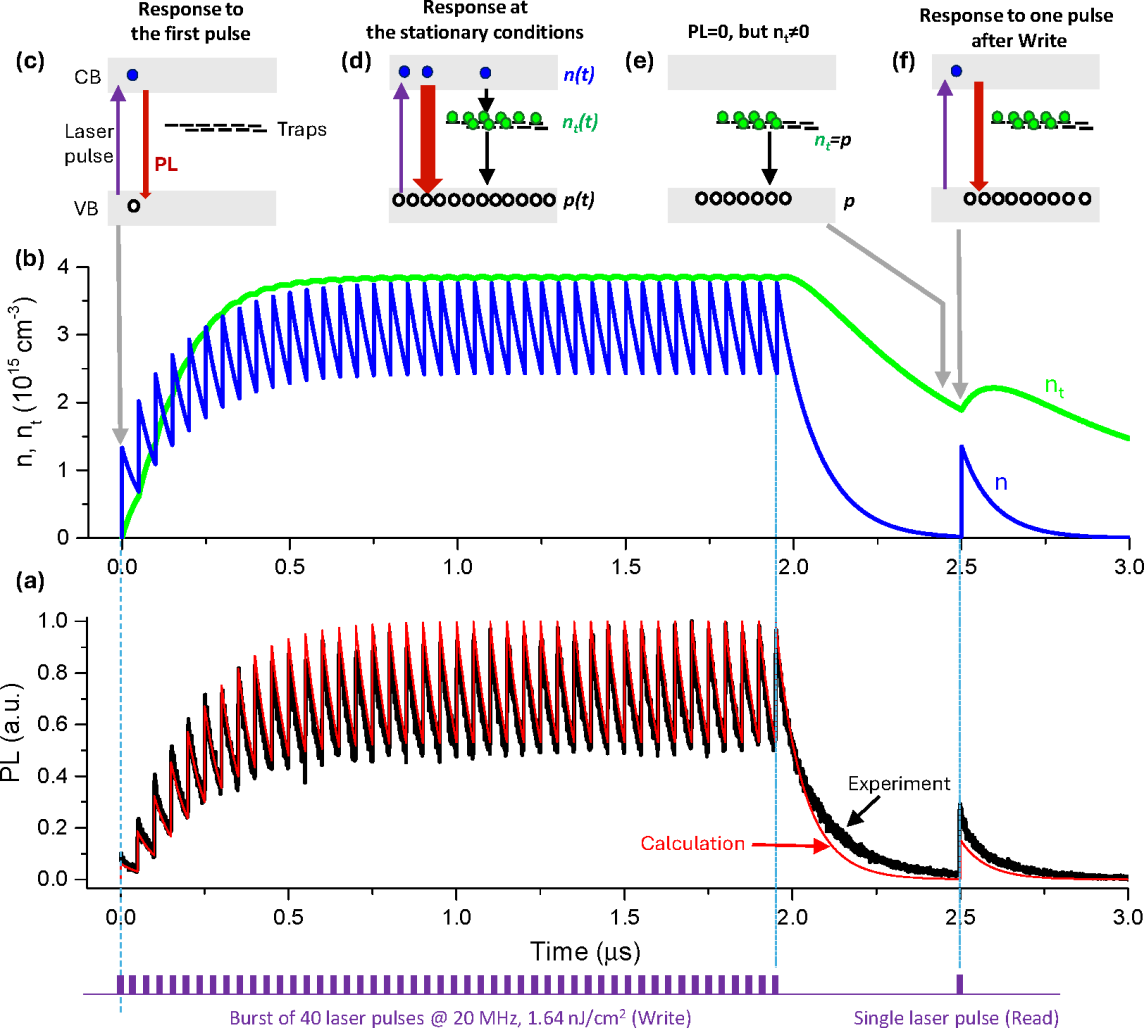
Demonstration of short-term memory in CsPbBr_3_ thin film memlumor. (a) The PL response of the memlumor under the pulse burst (Write) excitation followed by a single pulse (Read). The total repetition period of the pulse burst pattern equals 402.6 μs. The black curve of the experimental PL is plotted together with the SRH+ recombination model (red line; see Supplementary Note 5). The response of the single pulse after the burst is enhanced compared to the PL from the 1st pulse from the burst, signifying the presence of memory. (b) The modeled electron density (*n*) is plotted together with the density of trapped electrons (*n*_t_). Note that *n*_t_ just before the single Read pulse is not equal to zero, leading to the observed memory effect in PL. The dynamics of charge carriers under this type of excitation can be visualized by the schematic band diagrams (c–f). (c) At the beginning of the burst, the first laser pulse creates a concentration of electrons (*n*) and holes (*p*), while the concentration of trapped electrons (*n*_t_) equals to zero. (d) Under the equilibrium conditions of PL a certain value of *n*_t_ is built up. (e) Then after the end of the pulse burst the concentration of excited carriers starts to decay. The trapped electrons decay slowly so that before the single Read pulse their concentration is not zero. (f) Under the single Read pulse excitation, the PL is enhanced due to the presence of trapped electrons.

Intrinsic semiconductors (as MHPs mostly are) practically do not contain free electrons and holes under dark conditions (Figure 2c). When free charged carriers are generated, selected trapping of one type of them (e.g., electrons, Figure 2d) by defects results in the so-called photodoping effect—the excess of the concentration of one of the charge carriers (holes in our example) under light illumination. This occurs because of the charge neutrality *p* = *n* + *n*_t_, where *p*, *n*, and *n*_t_ are the concentrations of free holes, electrons, and trapped electrons, respectively. Because PL intensity is proportional to *p* × *n*, the increase of hole concentration due to photodoping increases the PL intensity (compare Figure 2c and Figure 2d).

Recombination of the trapped electrons (or relaxation of photodoping) is rather slow (microseconds) and inherently slower than that of the PL signal. So, the sample still contains substantial *n*_t_ when PL intensity is already close to zero (Figure 2e). Thus, the single-pulse PL response should be affected by the presence of trapped charges over their long lifetime, as we see experimentally (Figure 2f). This characteristic long lifetime of trapped charges in the microsecond range can be checked by the paired-pulse-facilitation experiment (Supplementary Note 4).

The charge carrier dynamics in the presence of traps discussed above can be described by a system of differential equations based on the Shockley–Read–Hall (SRH) charge recombination model (SRH+ model, Supplementary Notes 5.1, 5.2, 5.3, and 5.4) which was introduced previously:^41^
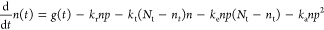
1
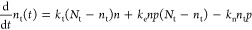
2
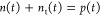
3
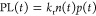
4where *g*(*t*) is the charge generation rate, *k*_r_ is the rate constant of radiative recombination, *N*_t_ is the concentration of electron traps, *k*_t_ is the rate constant of electron trapping, and *k*_n_ is the rate constant of trap depopulation by nonradiative recombination of trapped electrons with free holes. In addition, the third-order Auger processes are represented by Auger recombination with the rate constant *k*_e_ and Auger-assisted electron capture with the rate constant *k*_a_. Naturally, the PL(*t*) of a memlumor (eq 4) described by eq 1–eq 3 depends on the set of parameters above and time-dependent variables *n* and *n*_t_.

To use this model in practice, it is necessary to extract all of the model parameters for the particular sample investigated here. Among them, the most important parameters for the memory effects are *k*_r_, *k*_t_, *k*_n_, and *N*_t_. To quantify these parameters, we utilized the PLQY(*f*,*P*) mapping measurements discussed before (Figure 1b) combined with the PL decay kinetic measurements (Supplementary Notes 5.5 and 5.6). The data from these experiments were fitted using the SRH+ model with the obtained parameters summarized in Supplementary Table 4.

Using these parameters, we calculate the PL response to the Write–Read excitation pattern under our experimental conditions (red line in Figure 2a). The experimental (black) and predicted (red) curve match very well showing that for this sample and excitation conditions the simple SRH+ model with one trap type explains the behavior quite well. Also, Figure 2b shows the calculated concentrations of free and trapped electrons, quantitatively illustrating the previously discussed schemes in Figure 2c–f. Therefore, the processes described by the SRH+ model naturally explain the memory effects observed on the fast time scale, where the state vector ***X⃗*** = (*n*, *n*_t_, ...) changes with each individual excitation pulse. In other words, the variables *n* and *n*_t_ included in state vector ***X⃗*** define the response of the memlumor. Therefore, we can consider traps as memory cells, capable of remembering the history of light excitation.

There are also memory effects in CsPbBr_3_ memlumor occurring on time scales from ms to minutes.^36^ To perform an experiment showcasing the memlumor’s response on multiple time scales simultaneously, we combined a TCSPC technique with a nanosecond resolution with a measurement of the integrated PL signal on a CCD camera with a 100 ms resolution. The fast potentiation behavior of the CsPbBr_3_ film memlumor (left part of the Figure 3d) was measured using the TCSPC setup by applying a 20-pulse burst (80 MHz internal repetition frequency) with repetition period of 62.5 μs. Then, pulses at 80 MHz pulse repetition frequency were applied to study PL evolution on a CCD camera (right part of the Figure 3d). These two data sets are scaled to the same units using a calibration procedure: we assume that the stationary level reached at the end of the pulse burst (measured by TCSPC) is the same as the first data point measured by CCD (the first 100 ms of the experiment) when PL was excited by 80 MHz pulsed excitation. With this, we can track the evolution of PL response induced by the start of 80 MHz pulsed excitation at the time scale from ns to minutes (Figure 3d)

**Figure 3:**
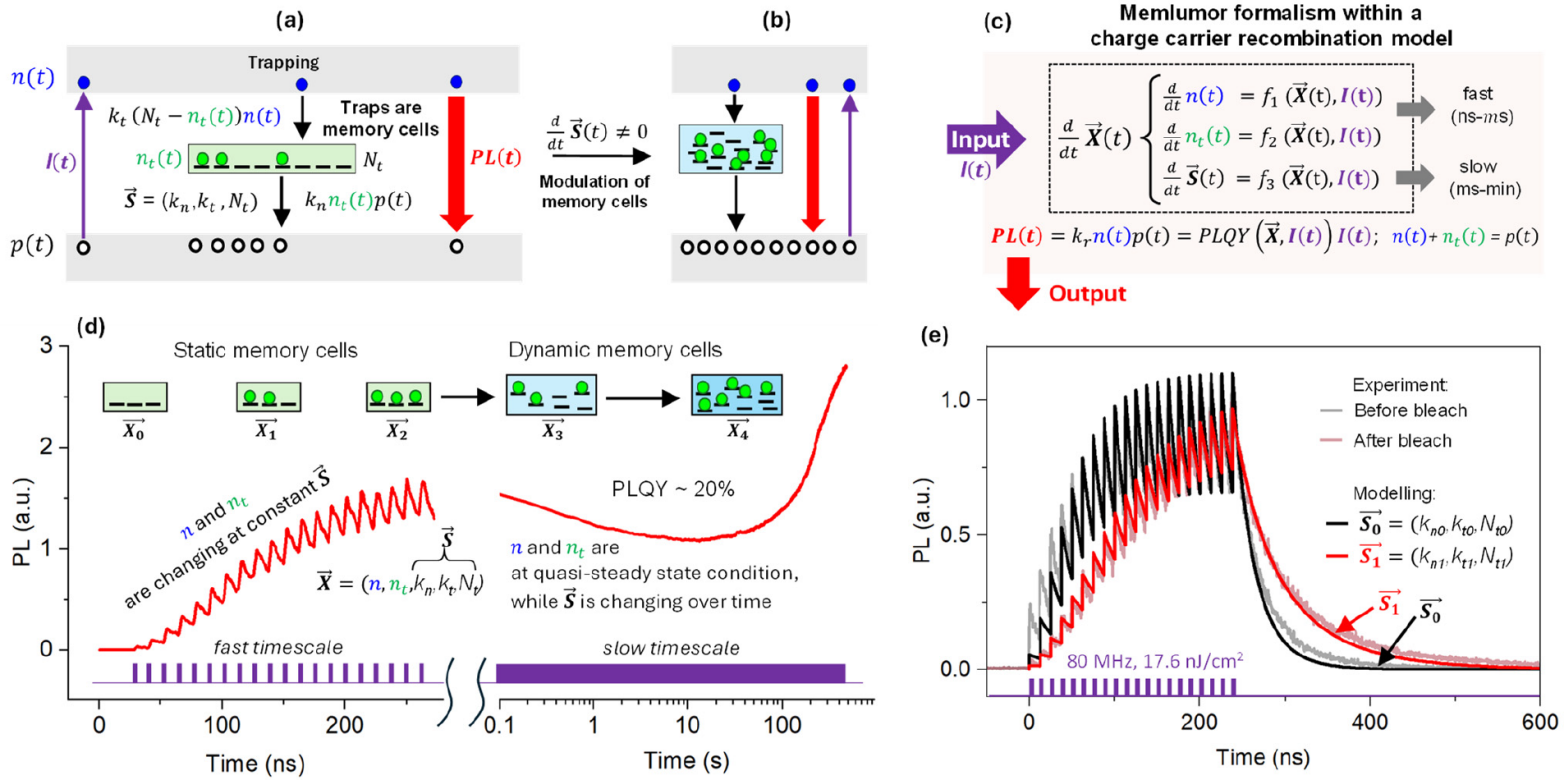
Physical processes behind the CsPbBr_3_ memlumor operation. (a) Band diagram explaining the charge dynamics under photoexcitation in the framework of the SRH recombination model. Trapping of electrons leads to photodoping and thus enhancement of PL. Traps can be seen as memory cells with the properties set by vector ***S⃗***(*t*) = (*k*_*t*_, *k*_*n*_, *N*_*t*_). (b) The properties of traps may change under prolong excitation. This transformation of traps changes ***S⃗***(*t*) which is different in (b) in comparison to (a). (c) Memlumor formalism in the framework of the SRH model. The PL intensity is determined by the set of differential equations on the memlumor state vector ***X⃗*** = (*n*, *n*_*t*_, *k*_*t*_, *k*_*n*_, *N*_*t*_). The fast (defined by functions *f*_1_ and *f*_2_) and slow (defined by function *f*_3_) components of the state vector can be separated. Dynamics of the fast components can be approximated by the SRH model (eqs 1 and 2) while the function *f*_3_ determining the dynamics of ***S⃗***(*t*) = (*k*_*t*_, *k*_*n*_, *N*_*t*_) is defined by material photochemistry. (d) Time-resolved PL response of the CsPbBr_3_ film under pulsed excitation monitored from ns to minutes. Excitation conditions: pulses 17.6 nJ/cm^2^ at 80 MHz, 1.4 W/cm^2^ average power density. The dynamics reflect the establishment of the quasi-equilibrated condition for the fast component (*n*, *n*_t_) of the state vector. Some of the memory cells become filled by electrons. However, at later times (seconds) the properties of the traps (memory cells) determined by the state vector ***S⃗***(*t*) = (*k*_*t*_, *k*_*n*_, *N*_*t*_) start to change, leading to the variation of the steady-state PL intensity. These changes are schematically illustrated by increasing the number of traps and changing their energy levels. (e) The PL responses to a burst of 20 laser pulses (80 MHz, 17.6 nJ/cm^2^) for the same CsPbBr_3_ film in two different states ***S⃗*_0_** and ***S⃗*_1_** are plotted together on the same scale with the fits obtained from the SRH+ model.

Importantly, while the SRH+ model describes the memlumor’s behavior on the fast time scale, it predicts that a quasi-steady state PL should be reached rather rapidly, typically within a few microseconds (Supplementary Note 5.3). On the other hand, our experimental results (Figure 3d, right part) indicate that that the state vector ***X⃗*** evolves on longer time scales (seconds), i.e., exhibiting a longer-term memory. The observation of this longer-term memory behavior makes it necessary to expand the model introduced above (Figure 3a) by including dynamics of the traps or, in other words, the time dependences *k*_t_(*t*), *k*_n_(*t*), and *N*_t_(*t*). Due to these dynamics, the memory cells of the perovskite memlumor should be considered as *dynamic memory cells* (Figure 3b).

This slow trap dynamics is intrinsic to MHPs and occurs as a consequence of slow photochemical processes leading to ion migration, material restructuring and thus to the evolution of defect properties.^21,51−55^ It is convenient to define a vector ***S⃗*** to represent the dynamic memory cell parameters: ***S⃗*** = (*k*_n_, *k*_t_, *N*_t_) (Figure 3b). Hence, the full state vector ***X⃗*** should contain both fast (*n*,*n*_t_) and slow (*k*_n_, *k*_t_, *N*_t_) changing variables, i.e. ***X⃗*** = (*n*, *n*_t_, ***S⃗***, ...) (Figure 3c,d). Thus, the generalized concept combining static and dynamic memory cells active across different time scales makes it possible to explain the PL(*t*) dependence shown in Figure 3d across the entire time frame from ns to minutes.

To summarize, the evolution of defect states under applied optical stimuli points to the coexistence of both volatile and nonvolatile memories across a wide range of times and energies in a semiconductor memlumor. Thus, the memlumor formalism (Figure 1a) can be described in the framework of the SRH+ model (eqs 1–4), with added slow dynamics of traps defined by the function *f*_3_ in Figure 3c.

Obviously, vector ***S⃗*** = (*k*_n_, *k*_t_, *N*_t_) determines not only the equilibrated value of PL at long time scales but also the response of the system to the input pulses at the nanosecond regime. This is illustrated in Figure 3e where the response of the sample to a writing burst of 20 pulses differs between the sample at an initial (pristine) sample state ***S⃗*_0_** and at a state ***S⃗*_1_**, which is achieved by preexposing the pristine sample to high light intensity illumination (bleaching). PL response of the CsPbBr_3_ memlumor in both states (***S⃗*_0_** and ***S⃗*_1_** ) is modeled theoretically by changing the vector ***S⃗*** in the SRH+ model (Supplementary Notes 5.5 and 5.6, Supplementary Tables 5 and 6).

Additional discussion about the vector ***S⃗***= (*k*_n_, *k*_t_, *N*_t_) impact on the memlumor response can be found in Supplementary Note 5.7. There we define the memlumor memory strength as a quantitative characteristic of the memory effect and calculate it for varied *k*_t_, *k*_n_, *N*_t_ and pulse fluence *P*. We found that the smaller pulse fluence makes the memory strength larger, while for given *N*_t_, *k*_n_, and *P*, changing the trapping rate constant *k*_t_ leads to a rather small change of the memory strength. However, the rate constant of trap depopulation *k*_n_ is found to strongly impact the memory. Thus, we can preliminarily conclude that the memlumor properties are very sensitive to the type of traps or, in other words, to their chemical nature and their position in the bandgap. Further efforts are needed to both theoretically and experimentally rationalize the influence of defects and their concentration on the memory strength to guide the further development of memlumors.

The realization of the memlumor concept offers a broad range of possibilities for its integration into neural networks due to the wealth of potential methods to address, couple, and read from these all-optical devices. For example, it is possible to employ them using free space coupling (Figure 4a), coupling by waveguides (Figure 4b,c) and coupling via near field. In the free-space regime, the excitation (input) and emission (output) light propagate in free space and can be delivered using optical elements (e.g., lenses) to a desired location with accuracy determined by the light diffraction limit. In practice, such design would lead to the limitation of the smallest addressable volume (size of an active element) to be approximately 1 cubic micrometer (Figure 4a). Note that the memlumor itself can be smaller; however, to address individual memlumors independently, the distance between them should be larger than the diffraction limit. Importantly, in such a scenario, there would be no need for wiring or interconnects since the connections are fully optical.

**Figure 4:**
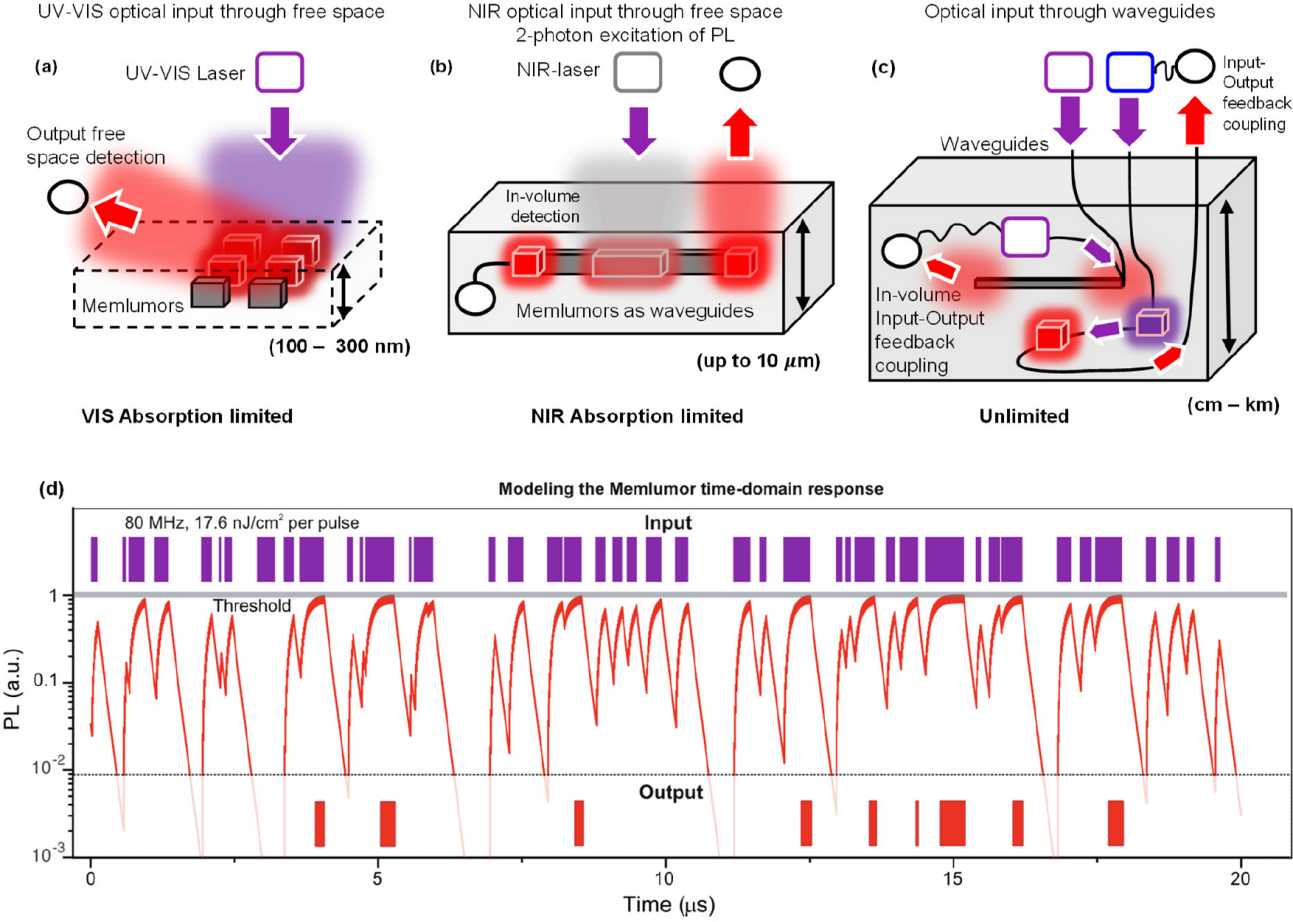
Possible applications of memlumors for in-memory calculations and neural networks. (a) 2D device with in-plane arrangements of individual memlumors addressed in far-field by free-space optics. (b) Quasi-3D device combining free-space input using 2-photon excitation, internal connections by waveguides, and output via free space to an external detector or via waveguiding to an internal detector. (c) Hypothetical 3D memlumor device using internal and external detectors coupled by waveguiding. (d) Calculated PL response (red) to the input signal presented by violet of the memlumor with the model parameters obtained for CsPbBr_3_ film (Supplementary Table 4). The output is not zero if the PL signal exceeds the threshold (gray line). Memlumor converts the violet barcode to the red barcode via the predefined signal threshold.

Moreover, for certain applications, it is not necessary to read each individual memlumor, and instead the total output of a certain group of devices (e.g., one row of a cross bar for matrix multiplication,^56^ or all memlumors together for summation operations) is of importance. These operations are natural, because the bosonic nature of photons allows the summation of the outputs from independent memlumors toward the same optical output detecting channel without their mutual interference. This promises wide parallelism in computing operations and the ability to continuously process online-updated data without information losses.

Another opportunity with memlumor systems is using the wavelength spectrum to tune the optical neuromorphic system response. For example, the fact that the PL energy is slightly red-shifted due to the Stokes shift opens additional opportunities for the integration of memlumors into three-dimensional (3D) architectures. This is because lower energy PL photons can propagate inside the semiconductor over a long distance without substantial attenuation. Thus, a fraction of the PL light generated in the depth of a thick crystal can still be detected by an external photodetector. This lifts the requirement that the devices have to be two-dimensional (2D) in nature (Figure 4b). Importantly, it is possible to address memlumors using a two-photon excitation with a near-infrared (NIR) laser, leading to a spatial resolution of 2 μm or better in the depth dimension (Figure 4b). We envision that a confocal microscope setup with one- and two-photon excitation capabilities and the point (confocal) and wide field excitation and detection modes is an ideal instrument for demonstrating and exploring the possibilities offered by memlumors for neuromorphic and optical calculations.

An alternative approach to addressing, reading, and coupling memlumors is based on the use of waveguiding (Figure 4b,c). MHPs exhibit high refractive indices making them excellent waveguiding materials. This makes it possible to combine the functionalities of memlumors with those of waveguides in the same material platform.

By showing such diversity of possible 2D and 3D integration, we consider spiking neural networks as the most perspective neural network architecture for the realization of memlumor-based photonic neuromorphic computing in the nearest future. In fact, having a wide time range of volatile physical mechanisms supported by nonvolatile memory, makes memlumors ideal material platform for such purpose. To illustrate the suitability of memlumors for the realization of spiking neural networks (SNN), we utilized the SRH+ model parameters extracted from the experimental data (Supplementary Table 4) to simulate the transformation of a time-dependent input to an output “barcode” by applying a detection threshold (which can be provided to the memlumor system by additional optical or optoelectronic components) (Figure 4d).

It is important to highlight that the practical realizations of systems based on memlumors do not require the costly TCSPC setup that was utilized here solely in order to investigate their photophysics. The input can be generated by utilizing inexpensive commercial pulsed laser diodes or even light-emitting diodes that are able to produce the desired pulse burst sequences in the time range from nanoseconds to microseconds with an amplitude modulation, while narrowband photodetectors could be used for detecting optical output. These requirements are already fully satisfied in standard optical communication systems, where the bandwidths in the range of up to tens of GHz are standard.

Some of the application ideas presented above can be realized experimentally using CsPbBr_3_ microcrystals as memlumors. For example, by attaching three CsPbBr_3_ microcrystals to a GaP waveguide (Supplementary Note 1),^57^ we are able to realize a summation operation (Figure 5a, Supplementary Note 6). Upon excitation, the response of all three memlumors is collected by the waveguide (because refractive index of GaP is 3.5 which is larger than that of CsPbBr_3_ (2.4))^57^ summed and detected at the edge of the waveguide. Another experimental demonstration relies on the use of a CsPbBr_3_ microwire that serves both as a memlumor and as a waveguide (Figure 5b). In this case, it is possible to tune the synaptic weight by changing the laser excitation position on the microwire. Importantly, the maximum value of the state-dependent PLQY can be up to 100% and still be controlled by the population of the defect states, ensuring an acceptable level of losses in optical schemes based on the memlumors. Even in our standard CsPbBr_3_ films the PLQY value is around 20% (Figure 3 d, Figure 1b).

**Figure 5:**
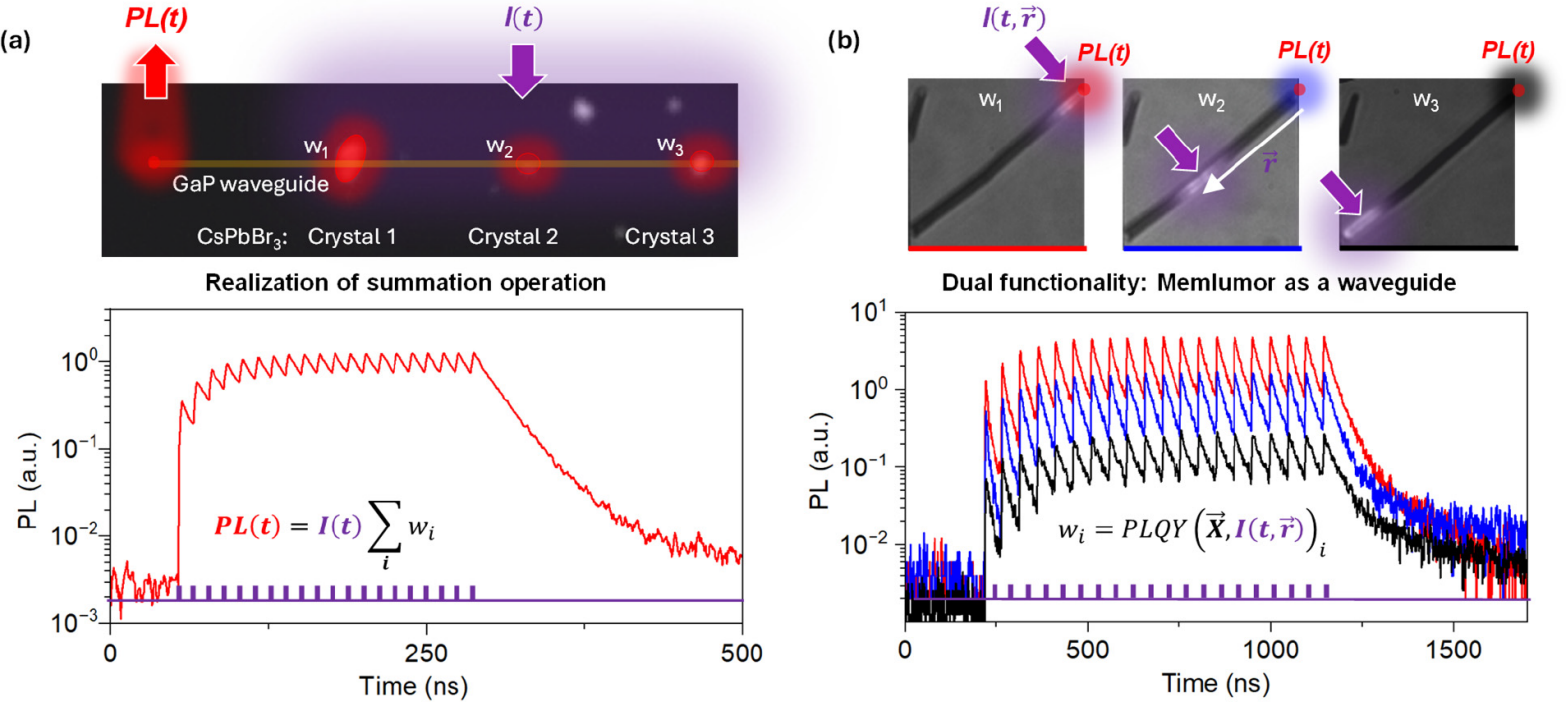
Examples of PL signal processing by memlumors. (a) Summation of the PL signal from three CsPbBr_3_ crystals coupled to a GaP waveguide by measuring PL from the end of the waveguide. Top panel: PL image of the structure with the PL from the crystals colored in red. Bottom panel: PL signal collected from the end of the waveguide when excited by a burst of 20 pulses. Here, the total PL signal is summed from all crystals with their synaptic weight *w*_*i*_. (b) Using a CsPbBr_3_ microwire as a spatially extended memlumor and the waveguide at the same time. Top panel: PL images of the CsPbBr_3_ microwire under three different locations of the focused excitation *I*(*t*, ***r⃗***). The horizontal image size is 30 μm. Bottom: PL signal collected from the end of the microwire (marked by a red spot in the image) for the three positions ***r⃗*** of the excitation beam.

To summarize, we show the memlumor concept using MHPs as a material platform that makes it possible to exploit their rich defect physics to have a direct impact on the memlumor’s properties. Specifically, the combination of photodoping and photochemistry phenomena makes it possible to utilize naturally existing traps in perovskite semiconductors as static and dynamic memory cells that define both volatile and nonvolatile memory effects on time scales ranging from ns to minutes.

By elucidating the fundamental photophysical principles, we demonstrate that the description of semiconductor memlumors using the Shockley–Read–Hall-based model suggests their state vector ***X⃗*** to contain both fast (*n*, *n*_t_) and slow (*k*_n_, *k*_t_, *N*_t_) changing variables. This makes memlumors particularly promising for further integration and application in developing neuromorphic computing by using spiking neural network architectures.

Finally, while our work provides the conceptual framework of memlumors, examines their fundamental operational physical mechanisms, and offers several examples for their applications, significantly more research is required in order to realize memlumor-based neural networks. This includes the large-scale system integration of memlumors based on near-field or/and far-field optics, the development of novel algorithms for energy-efficient computing, and the realization of on-chip and 3D integrated memlumors in existing photonics neuromorphic components and combining with other neuromorphic photonics technologies such as phase change memories. These examples illustrate how the novel concept of a memlumor will drive innovation in a range of research fields working together toward a gradual transition into next-generation neuromorphic computing paradigm.

## Supporting Information Available

Synthesis of the materials for memlumors (Supplementary Note 1), optical setup and methods for memlumor characterization (Supplementary Note 2), description of the PLQY (f,P) mapping for the CsPbBr_3_ thin film memlumor (Supplementary Note 3), paired pulse facilitation of CsPbBr_3_ and MAPbI_3_ memlumors (Supplementary Note 4), theoretical modeling and calculations (Supplementary Note 5), and CsPbBr_3_ memlumor crystals integrated on GaP waveguides (Supplementary Note 6) (PDF)
