# Supplementary material for: Charge Trapping and Defect Dynamics as Origin of Memory Effects in Metal Halide Perovskite Memlumors
Source: J Phys Chem Lett. 2024 Jun 6;15(24):6256–65. doi: 10.1021/acs.jpclett.4c00985 (PMC11197924; doi:10.1021/acs.jpclett.4c00985)
Supplement: Supplementary file 1 — jz4c00985_si_002.pdf [file jz4c00985_si_002.pdf]

Supplementary information for

Charge Trapping and Defect Dynamics as Origin of Memory Effects in Metal Halide Perovskite Memlumors

Alexandr Marunchenko[1*], Jitendra Kumar[1], Alexander Kiligaridis[1], Shraddha M. Rao[1], Dmitry Tatarinov[2], Ivan Matchenya[2], Elizaveta Sapozhnikova[2], Ran Ji[3,4], Oscar Telschow[3,4], Julius Brunner[3,4], Alexei Yulin[2], Anatoly Pushkarev[2], Yana Vaynzof[3,4], Ivan G. Scheblykin[1*]

*Corresponding author(s). E-mail(s): a.marunchenko@metalab.ifmo.ru; ivan.scheblykin@chemphys.lu.se;

[1] Chemical Physics and NanoLund, Lund University, P.O. Box 124, 22100 Lund, Sweden

[2] School of Physics and Engineering, ITMO University, 49 Kronverksky, St. Petersburg 197101, Russian Federation

[3] Chair for Emerging Electronic Technologies, Technical University of Dresden, Nöthnitzer Str. 61, 01187 Dresden, Germany

[4] Leibniz-Institute for Solid State and Materials Research Dresden, Helmholtzstraße 20, 01069 Dresden, Germany

Table of content

Supplementary Note 1.
Synthesis of Materials for Memlumors 3
1.1 $CsPbBr_3$ perovskites 3
1.2 Other metal halide perovskites 4
.
Supplementary Note 2.
Optical Setup and Methods for Memlumor Characterization 6
2.1 Optical setup 6
2.2 Light excitation conditions 7
2.3 Measurements of time-resolved PL under pulsed burst excitation using TCSPC 8
2.4 PLQY(f,P) mapping 10

Supplementary Note 3.
Description of the PLQY (f, P) Mapping for the $CsPbBr_3$ Film Memlumor 10

Supplementary Note 4.
Paired Pulse Facilitation of $CsPbBr_3$ and $MAPbI_3$ Memlumors 16

Supplementary Note 5.
Theoretical Modeling and Calculations 17

5.1. Shockley-Read-Hall (SRH) model with added radiative recombination (SRH+radiative) 17
5.2. Extended Shockley-Read-Hall model (SRH+) with added Auger processes 18
5.3. Pulsed photoexcitation in the framework of the SRH+ model 19
5.4 Solution of the model equations of SRH+ radiative model in the special case of low excitation fluence 21
5.5 Modelling protocol for the extraction of the SRH+ model parameters 23
5.6. Extracting parameters according to modeling protocol. Fitting experimental data for the $CsPbBr_3$ film memlumors 24
5.7 Modelling of the short-term memory effect by modifying parameters of trap states 25

Supplementary Note 6.
$CsPbBr_3$ Memlumor Crystals Integrated on GaP Waveguides 28

References 29

Supplementary Note 1. Synthesis of Materials for Memlumors

1.1 $CsPbBr_3$ perovskites

Materials:
Cesium bromide (CsBr, 99.99%, TCI Chemicals), cesium carbonate (Cs_2CO_3, 99%, Sigma-Aldrich), lead(II) bromide ($PbBr_2$, 99.999% trace metals basis, TCI chemicals), dimethyl sulfoxide (DMSO, anhydrous ≥99.8 %, Sigma-Aldrich), diphenyl ether (DE, ≥99%, Sigma-Aldrich), oleic acid (OA, technical grade, 90%, Sigma-Aldrich), oleylamine (OLAm, technical grade, 70%, Sigma-Aldrich) were used as received.

Preparation of perovskite precursor solution:
CsBr (62 mg) and $PbBr_2$ (110 mg) were mixed and dissolved in DMSO (1 mL) by shaking without heating to obtain a clear 0.3M solution. The solution was filtered through a 0.45 μm PTFE syringe adapter right before the procedure of perovskite film deposition.

$CsPbBr_3$ polycrystalline film preparation:
The glass substrates (15 x 15 mm^2) were cleaned by sonication in $NaHCO_3$ solution, deionized water, acetone, and 2-propanol for 10 min consecutively, and then exposed to UV-generated ozone for 15 minutes to obtain a hydrophilic surface. Afterwards, substrates were transferred in the dry glovebox filled with N_2 gas. The deposition of perovskite films was conducted on the substrates by single-step spin-coating method at 3000 rpm for 5 minutes. Thereafter, the samples were gradually annealed on a hot plate from 50 °C up to 130 °C for 15 min to remove dimethyl sulfoxide residues and complete the crystallization of perovskite.

$CsPbBr_3$ microwires preparation:
For the $CsPbBr_3$ microwires (MWs) synthesis, the temperature difference-triggered growth method was used.[1] For this, a furnace (PZ 28-3TD High-Temperature Titanium Hotplate and Program Regler PR5-3T) was employed to control the temperature during the MWs growth. The $CsPbBr_3$ perovskite material was sublimated from a source substrate to a target substrate. As a source substrate, the $CsPbBr_3$ polycrystalline film (prepared by the method described above) was used. The target sapphire substrate (10 x 10 mm^2) was cleaned by sonication in deionized water, acetone, and 2-propanol for 10 min consecutively. Two substrates were separated with an air gap of 0.5 cm. The temperature of both substrates was controlled by the furnace temperature. The synthesis was initiated at a furnace temperature of 350 °C. Afterwards, the temperature was increased up to 520 °C for 10 min and kept unchanged for 10 min. As a result, the sublimated $CsPbBr_3$ microwires were grown in three crystallographic directions of the sapphite.

$CsPbBr_3$ NPs on GaP nanowaveguides preparation:
Cs_2CO_3 (0.407 g) was loaded into a 100 mL flask along with octadecene (20 mL) and oleic acid (OA, 1.25 mL), dried for 1 h at 120 °C, and then heated up to 150 °C to give a clear CsOA 0.125M solution. All manipulations were conducted in a N_2-filled glove box. Perovskite nanoparticles (NPs) were synthesized using a Schlenk line by modified hot-injection method. $PbBr_2$ (35 mg) was added to a 50 ml two-neck flask and dried in DE solvent at 120 °C under vacuum for 40 min. Then, OA (150 μL) and OLAm (150 μL) were added dropwise to it. Thereafter, the flask was purged with N_2 and the mixture was heated up to 150 °C to obtain a clear solution. The solution was cooled down to 88°C before the injection of preheated at 120°C CsOA solution (0.5 mL) followed by incubation for 2 h at 88 °C. Afterwards, the solution was

heated up to 150 °C, incubated for 30 min, and, finally, quenched by using an ice bath. The product was centrifuged at 3000 rpm for 5 min, separated from supernatant solution, and redispersed in 10 mL of n-hexane. Large particles were settled down in 5 min and upper fraction of the solution (3 mL) was pipetted for further manipulations. This fraction contains a wide dispersion of NPs with the mean size of about 360 nm according to dynamic laser scattering measurements.

GaP NWs were grown on a Si substrate by molecular beam epitaxy using a protocol reported by Trofimov et al.[2] NWs were transferred into suspension by ultrasonication of 0.5x0.5 cm substrate in 1 mL of 2-propanol, drop-casted onto a glass substrate, and rinsed with hot acetone two times. Then, the solution containing $CsPbBr_3$ NPs was drop-casted over GaP NWs to give nanowaveguides decorated with subwavelength light emitters.

1.2 Other metal halide perovskites

$MAPbI_3$ and $MAPbI_{2.96}$ films:
MAI and $Pb(Ac)_2{\cdot}3(H_2O)$ (at 3:1 or 2.96:1molar ratio) were dissolved in anhydrous *N,N*-dimethylformamide (DMF) with a concentration of 40 wt% with the addition of hypophosphorous acid solution (6 µL / 1 mL DMF). The perovskite solution was spin coated at 2000 rpm for 60 s in a drybox (RH < 0.5 %). After spin coating, the samples were dried for 20 seconds by a stream of dry air. Afterwards, the samples were kept at room temperature for 5 min and subsequently annealed at 100 °C for 5 min.

Triple cation perovskite films:
The perovskite precursor solution was prepared by dissolving PbI_2 and $PbBr_2$ in a solvent mixture (DMF/DMSO = 4/1) and CsI in DMSO at 180 °C for 10 minutes. After cooling down, CsI, PbI_2 and $PbBr_2$ solutions were mixed in a volume ratio of 0.05:0.85:0.15, to obtain an inorganic stock solution. MAI and FAI powders in separate vials were then dissolved to form stock solutions. Finally, the MAI-stock-solution was mixed with the FAI-stock-solution in a 1:5 volume ratio to acquire the final 1.2 M $Cs_{0.05}(FA_{5/6}MA_{1/6})_{0.95}Pb(I_{0.9}Br_{0.1})_3$ perovskite solution in DMF and DMSO. The perovskite layer was deposited via a two-step spin-coating procedure with 1000 rpm for 12 s and 5000 rpm for 28 s. Before spinning, 40 µL of perovskite precursor solution were applied to the sample statically and 150 µL of Trifluorotoluene (TFT) was dripped on the spinning substrate, 5 s before the end of the second spin-coating step in a rapid fashion, in agreement with Taylor et al.[3] Subsequently, the samples were annealed at 100°C for 30 min.

$CsPbI_3$ QDs perovskite films:
The synthesis of the perovskite QDs was done via the hot injection method, firstly published by Protesescu et al., with several adjustments.[4] Before the synthesis, oleic acid (OA, technical grade 90%, Sigma Aldrich) and oleylamine (OLA, technical grade 70 %, Sigma Aldrich) were degassed at 100 °C for 1 h to guarantee high purity of the reactants. Cs-oleate was produced by combining Cs-carbonate with OA. For the preparation of Cs-oleate solution, 0.407 g of Cs_2CO_3 (TCI, >98 %), 20 mL of octadecene (ODE, technical grade 90 %, Acros Organics), and 1.25 mL of OA were loaded in a 2-neck round-bottom flask and degassed for 1 h at 100°C in vacuum. Thereafter, the flask was filled with nitrogen and heated to 150 °C until all reactants reacted and a clear solution of Cs-oleate was obtained. The Cs-oleate was then stored in nitrogen at 70 °C until usage. For the synthesis of the $CsPbI_3$, 1 g of PbI_2 (99.99 %, TCI) and 60 ml of ODE were filled in a 2-neck round-bottom flask and degassed for 1 h at 120 °C in a vacuum. Subsequently, the flask was filled with nitrogen and 6 mL of OLA and 6 ml of OA

were mixed in a vial and then injected. The flask was again pumped to vacuum for 30 min, until a yellow transparent solution was obtained. Then, the flask was filled with nitrogen and heated to 170 °C. At the target temperature, 4 mL of Cs-oleate was quickly injected into the flask. The solution turned dark red and after 5 s the reaction was quenched with an ice-water bath. For the purification of the as-prepared $CsPbI_3$ nanocrystals, 12.5 mL of the crude solution was mixed with 37.5 mL of Methyl acetate (MeOAc, 99 %, Acros Organics) and centrifuged for 10 min at 6000 rpm. The supernatant was discarded and the wet $CsPbI_3$ pellets were redispersed in 3 mL of hexane (97%, Acros Organics). The solution was again mixed with 5mL of MeOAc and centrifuged for 10 min at 6000 rpm. The supernatant was removed, and the precipitates of all tubes were combined in one and dispersed in 25 mL of hexane. This solution was centrifuged for 5 min at 4000 rpm and this time the supernatant was collected and stored overnight at 4 °C. After that, the solution was centrifuged again for 5 min at 4000 rpm. Finally, the supernatant was collected and dried by using a rotary evaporator. The obtained $CsPbI_3$ nanocrystals were dispersed in octane (99%, Acros Organics) at a concentration of 75 mg/mL for further use.

Then, for fabrication of $CsPbI_3$ quantum dot samples we followed the procedure reported previously[5] with slight modifications. Glass substrates were cleaned thoroughly by sonicating them in acetone and isopropanol for 15 min respectively. To activate the surface and remove organic remains the samples were subjected to oxygen plasma for 10 min. After that, they were transferred to a nitrogen-filled glovebox for the deposition of the perovskite quantum dots. The quantum dot solution that was obtained from the synthesis was then spin-coated dynamically on the glass substrates at a speed of 1000 rpm for the first 5 s and 2000 rpm for the following 10 s. To exchange the long organic ligands, the film was in immersed in a ligand solution of sodium acetate (NaOAc, 99.995 %, Sigma Aldrich) in MeOAc for 5 s and was spin-dried, followed by soaking and spin-drying the film in MeOAc for 5 s twice. This procedure was repeated four times to obtain a layer of approximately 250 nm. After that the sample was soaked in a solution of phenethylammonium iodide (PEAI, Great Cell Solar) in ethyl acetate for 10 s and spin-dryed. The PEAI treated sample was then washed with ethyl acetate.

PMMA layer deposition:

The PMMA coating of all different types of perovskite films was performed by preparing a solution of polymethyl methacrylate (PMMA, Greatcell Solar) in chlorobenzene (10 mg/ml) that was then spin-coated at 3000 rpm for 30 s on top of each of the layers.

Supplementary Note 2.
Optical setup and Methods for Memlumor Characterization

2.1 Optical setup

The same optical setup as described in the previous publications[6,7] was used. The setup is based on a home-built wide-field photoluminescence microscope with Olympus IX71 as the core. 40X (NA=0.6) dry objective lens and an EMCCD Camera (ProEM, Princeton Instruments) as the primary detector are used. The PL was excited by a 485 nm diode laser (PicoQuant, 200 ps pulse width) through the objective lens. The laser is controlled by the laser driver SEPIA LD828 (PicoQuant). This allows to obtain at any pulse repetition rate from 80 MHz to 0.2 Hz for the laser pulses. Also, the same controller allows for a pulse burst mode and even more complex pulse patterns. The setup is equipped with software-controlled shutter and several neutral density filter wheels to adjust the pulse fluence (P_i) of the laser and to attenuate the PL light sent to the detectors in automatic regime. In addition to detection by the camera, the PL signal can be sent to the hybrid photomultiplier detector (HPD, Picoquant PMA Hybrid-42) connected to a time correlated single photon counting (TCSPC) module (Picoquant, Picoharp 300) for measurements of PL decays kinetics. The instrumental response function time width is approximately 200 ps.

All components of the setup are controlled by a home developed Labview software. This software allows to execute a designed sequence of measurements (recording a PL image with the camera or a PL decay curve) according to a list of instructions (table with experimental parameters like OD of the filters, exposure time, laser repetition rate and so on). This automatic measurement mode allows to perform complex measurements like PLQY(f,P) mapping, see **Supplementary Note 2.4 and 3** .

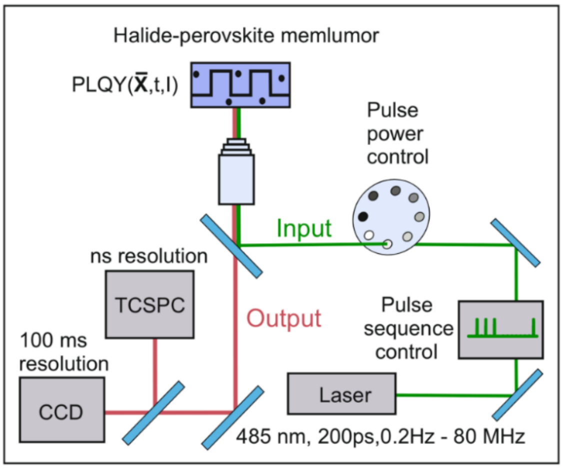

Supplementary Fig. 1. The experimental setup used for characterization of memlumors, also described elsewhere.[6]

2.2 Light Excitation Conditions

Excitation conditions and values of the induced charge carrier densities were the same as described in our recent publication.[6,8]

The initial value of the photogenerated charge carrier density n_0 can be estimated for each of the pulse fluence values P_i as $n_0 = \frac{P_i \cdot Abs}{d}$, where d is the film thickness and Abs in the film absorption. $Abs = (1 - S)(1 - R)\,(1 - e^{-\alpha d})$, where scattering S<<1, R is reflectance and α is the absorption coefficient of the material. In the simplest case R = $\frac{(n-1)^2}{(n+1)^2}$, where n is refractive index of a material at a corresponding wavelength.

For the $CsPbBr_3$ polycrystalline thin film we consider $n \approx 2.3\,, \alpha \approx 0.45 \cdot 10^5\ cm^{-1}$ at a 485nm wavelength.[9,10] Thus, for film thickness of 90 nm, Abs=0.28. Therefore, n_0 for each of the pulse fluence P_i is equal to:

$$n_0 = P_i \cdot 0.31 \cdot 10^5\ cm^{-1}$$

The calculated values for n_0 are listed in **Supplementary Table 1** below.

Supplementary Table 1. Values of the five different pulse fluences P1-P5 mainly used in the experiments, the corresponding charge carrier densities n_0, pulse energy densities, and average power densities. The color scheme shown in the table is used in **Fig. 1b** of the paper.

Pulse fluence P_i (photons/cm^2)	Charge carrier density n_0 (cm^{-3})	Pulse energy density, (nJ/cm^2)	Average power density at 80 MHz repetition rate (W/cm^2)
**P5 = 4.9x10^{12}**	**1.52x10^{17}**	**2000**	**160**
**P4 = 5.4x10^{11}**	**1.67x10^{16}**	**220**	**17.6**
**P3 = 4.3x10^{10}**	**1.33x10^{15}**	**17.63**	**1.41**
**P2 = 4.0x10^{9}**	**1.24x10^{14}**	**1.64**	**0.131**
**P1 = 3.4x10^{8}**	**1.05x10^{13}**	**0.14**	**0.0113**

2.3 Measurements of time-resolved PL under pulsed burst excitation using TCSPC

In our experiments we used a standard time-correlated single photon counting (TCSPC) setup in a non-standard regime. The setup is based on a picosecond diode laser, multichannel picosecond diode laser driver SEPIA 828 (PicoQuant) and time counting device PicoHarp 300 (PicoQuant).

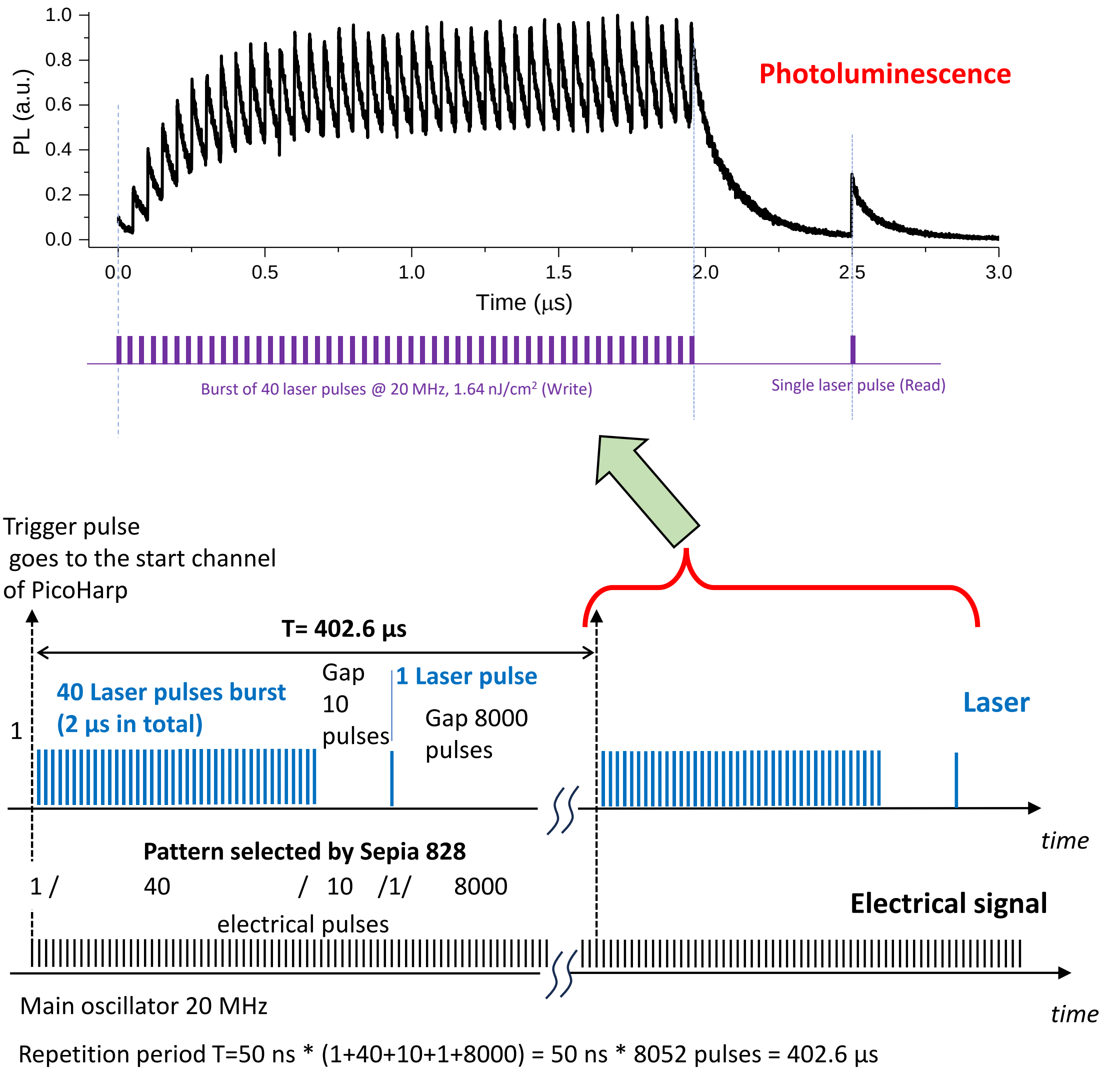

Supplementary Fig. 2. TCSPC technique with excitations by a burst of laser pulses.

In the standard TCSPC scheme, the sample is excited by a pulsed laser with a fixed repetition rate. For example, if the repetition rate is 100 kHz, sample is excited each T=10 µs. For each period T, a trigger signal from the laser driver arrives to the first channel (start pulse) of the time TCSPC device. After detecting this signal, the device measures the time until the stop pulse arrives to its second channel from the photodetector (one detected photon generates an electrical pulse). The histogram of the delays between the start and the stop pulses builds over time and shows the shape of the PL decay.[11]

Contrary to this standard regime, the memlumors are excited by a burst of several closely separated laser pulses and this burst is repeated with a repetition period T. Such pulse time-pattern is created by a Sepia 828 oscillator triggering the laser diode. For example, the experiment shown in **Fig. 2a** was carried out using the following time sequence of the pulses per one period T (see also **Supplementary Fig.2**):

The basic frequency was set to 20 MHz resulting in a 50 ns separation between the pulses. The entire pulse sequence is the following:

- The 1st pulse creates the start signal for the TCSPC electronics;
- The next 40 pulses are sent to the laser driver and produce the burst of 40 laser pulses which excites the sample;
- The next 10 pulses are skipped creating a delay of 550 ns;
- The next single pulse is sent to the laser driver and produces one laser pulse which also excites the sample;
- The next 8000 pulses are skipped to create a large delay.

The entire sequence is repeated over 100 s to accumulate the PL response curve.

The total time of the sequence consists of 1+40+10+1+8000 = 8052 pulses and takes 8052×50 ns = 402.6 μs corresponding to the repetition frequency of 1/402.6 μs = 2484 Hz.

We would like to stress several important practical issues concerning the possible photon count rates in such an excitation scheme. Older generations of TCSPC electronics operated in such a way that after detection of a stop signal the system was waiting until the next start pulse to start the counting again. Thus, not more than just one stop pulse could be detected. As a consequence, in order to obtain a histogram which would reflect the real PL decay curve without disturbance by the pile-up effect, one needed to limit the number of stop pulses per start to a value that was much less than one. The practical "rule of thumb" was to set the probability to detect a stop photon less than 5% per an excitation pulse. Thus, the photon counting rate was < 5 % of the repetition rate of the laser pulses.

If we would follow this rule with the burst excitation scheme discussed above, the maximum count rate of the signal should be limited to 5%×2488 Hz= 124 counts per second only. Note that the dark counts of the detector (PMA Hybrid detector, PicoQuant) is approximately 200 s^{-1}. Thus, with this limited detection count rate it is not possible to obtain a curve with a high signal to noise ratio for low excitation intensities, such as the one presented in **Supplementary Fig.2**. However, fortunately, this rule is not applicable to the experimental conditions applied to the memlumors. As we will explain in the following, a much higher signal count rates can be used.

This is possible because the current generations of TCSPC systems are able to count the time difference relatively to the start pulse for many detection events per repetition period as long as the photons do not arrive during the detection system dead time (each photon detection event locks the system for the deadtime). So, the pile-up effect in its classical sense is only present when the repetition period of the laser pulses or the fluorescence decay itself is shorter than the deadtime. For example, for our system (PicoHarp 300) the deadtime is approximately 100 ns. Based on this, only for the laser repetition rates larger than 1 MHz (time gap to the next pulse is < 100 ns) does the 5% rule apply.

In the experiments with memlumors, the repetition rate is as low as 2.5 kHz (400 μs repetition period). At the same time, the PL signal is spread over the time window of approximately 3 μs. These times are substantially larger than the detection deadtime (100 ns). Therefore, several photons can be detected per repetition period without any pile-up effect. This leads to a count rate of several kHz, which is much larger than the noise counts. The maximum safe count rate depends on the shape of the PL response curve. In practice, test experiments should be performed by increasing the count rate to identify the point when a distortion of the PL response due to the pile up effect becomes visible. Then a rate of at least 10 times smaller should be used.

2.4 PLQY(f,P) mapping

For the PLQY(f,P) mapping experiments[7,12] the sample is excited by a pulsed laser at repetition rate f [s^{-1}] and pulse fluence P_i [photons/cm^2] which are controlled by the laser driver and a neutral density filter wheel, respectively. The sample PL intensity is measured for each combination of f and P. We used a CCD camera to measure the PL integrated over 30 μm laser excitation spot. Because the entire system is calibrated, the PL intensity can be converted to an external PLQY.[7,12] To acquire the PLQY(*f*,P) map, PL is measured for a laser excitation spanning over 4 orders of magnitude (from *ca.* 10^8 to 10^{12} photons/cm^2/pulse) in 4 steps with power fluences P_i - P1, P2, P3, P4 and P5 (each step changes the fluence approximately 10 times, see **Supplementary Table 1**) and almost 7 orders of magnitude in pulse repetition rate, i.e., from 10 Hz to 80 MHz (**Supplementary Table 2, Supplementary Table 3)**. For each pulse fluence P, the repetition rate, *f*, is scanned across the entire range. Usually, a complete map consists of 50-150 data points.

All the data points of the PLQY(*f*, P) map are acquired automatically because the setup is fully controlled by home-developed LabVIEW program. It executes the experiment according to a pre-loaded table of parameters for the data acquisition (*f*, P, shutter timing, acquisition time of the camera, filters, *etc.*). To minimize the sample exposure, the shutter is synchronized with camera acquisition to allow the laser beam to irradiate the sample during the PL acquisition only. The program automatically saves the PL images, which are later processed using another program to yield the PLQY(*f,P*) map. This ensures that the data acquisition conditions are fully reproducible to be able to repeat exactly the same experiment with another sample. This is of curial importance for light-sensitive materials like metal halide perovskites and memlumors in general. The complete measurement takes from 1 to 3 hours, where the longest time is required to acquire data for low P and low f values since exposure times as long as several minutes per data point are often essential.

Supplementary Note 3. Description of the PLQY (f, P) Mapping for the $CsPbBr_3$ Film Memlumor

The goal of the PLQY(f,P) mapping is to obtain the parameters of the defect states $\vec{\boldsymbol{S}} = (k_t, k_n, N_t)$ and all other rate constants of the SRH+ model by probing the PL upon various excitation conditions. This changes the components of the state vector responsible for the short-term memory (n, n_t) over a broad range. Note that because these components react to any change of the excitation conditions very fast (much faster than a microsecond), the PL is always measured at the quasi-steady state conditions for (n, n_t) (see **Supplementary Note 5.3**) under the standard experimental conditions[7] when the exposure time per data point (a

combination of f and P) is larger than 1 ms. So, PLQY(f,P) mapping makes it possible to obtain the components of the state vector $\vec{\boldsymbol{S}} = (k_t, k_n, N_t)$ assuming that these components are not affected by the experimental procedure, which mathematically means that:

$$\frac{d\overrightarrow{\boldsymbol{S}(f,P)}}{dt} = 0 \qquad \text{S3.1}$$

However, this assumption contradicts the knowledge of $CsPbBr_3$ as a photosensitive material, where the parameters of the defect states are light sensitive. In other words, it is known that the experiment itself may influence the result of the measurement (the so-called observer effect, see details in reference[12]). Considering that the PLQY(f,P) measurements involve both very low and very high excitation power densities, extra care should be taken so that the results are not affected by a constant evolution of the material under light irradiation (excitation history). This means it is important to consider that the same data point measured with different irradiation time (exposure time) might result in a different value of PLQY.

Supplementary Fig.3a shows the PLQY(f,P) map obtained for the $CsPbBr_3$ film studied here using the standard experimental protocol used previously for these type of measurements.[7] The parameters used to measure each experimental point are shown in **Supplementary Table 2**. A simple visual inspection of the PLQY(f,P) plot reveals the presence of a significant sample instability.[12] This is evidenced by the appearance of a non-constant PLQY where the single pulse excitation regime is expected and the absence of a common quasi-CW regime for all the pulse fluences (see **Supplementary Note 5** and references[7,12] as well as the labels in **Supplementary Fig.3a**).

To understand this, it is helpful to roughly split the entire PLQY(f,P) map into two regions: **Region 1**, in which the averaged excitation power density is mild or low and **Region 2**, in which the averaged power density is high. **Supplementary Table 2** lists the experimental parameters for the measurements. The vector $\vec{\boldsymbol{S}} = (k_t, k_n, N_t)$ is likely to change in Region 2, however the impact of this change is more clearly observed in Region 1, most likely due to the fact that non-radiative recombination is dominant in this region. Hence, to track the change in the state of the memlumor during the measurements, a reference point is measured at a fluence P_2 at 80 MHz (inside Region 1) after each point of the PLQY (f,P) map.

The PL intensity at this reference point should be constant if the sample is stable, however, this is clearly not the case as can be clearly seen in **Supplementary Fig.3b**. The first and the last value of the reference point varies by two orders of magnitude. The evolution of the reference point (**Supplementary Fig.3b**) shows that entering Region 2 influences the sample significantly. Hence the values of the $\vec{\boldsymbol{S}}$ vector for Region 1 and Region 2 must be very different, and as a result, the PLQY(f,P) map is affected by the sample scanning history and cannot be fitted correctly because equation S3.1 does not hold ($\frac{d\overrightarrow{S(f,P)}}{dt} \neq 0$). Indeed, the shape of the PLQY (f,P) map contains features that are not compatible with any charge recombination model with constant parameters (see further explanation in **Supplementary Fig.3a**).

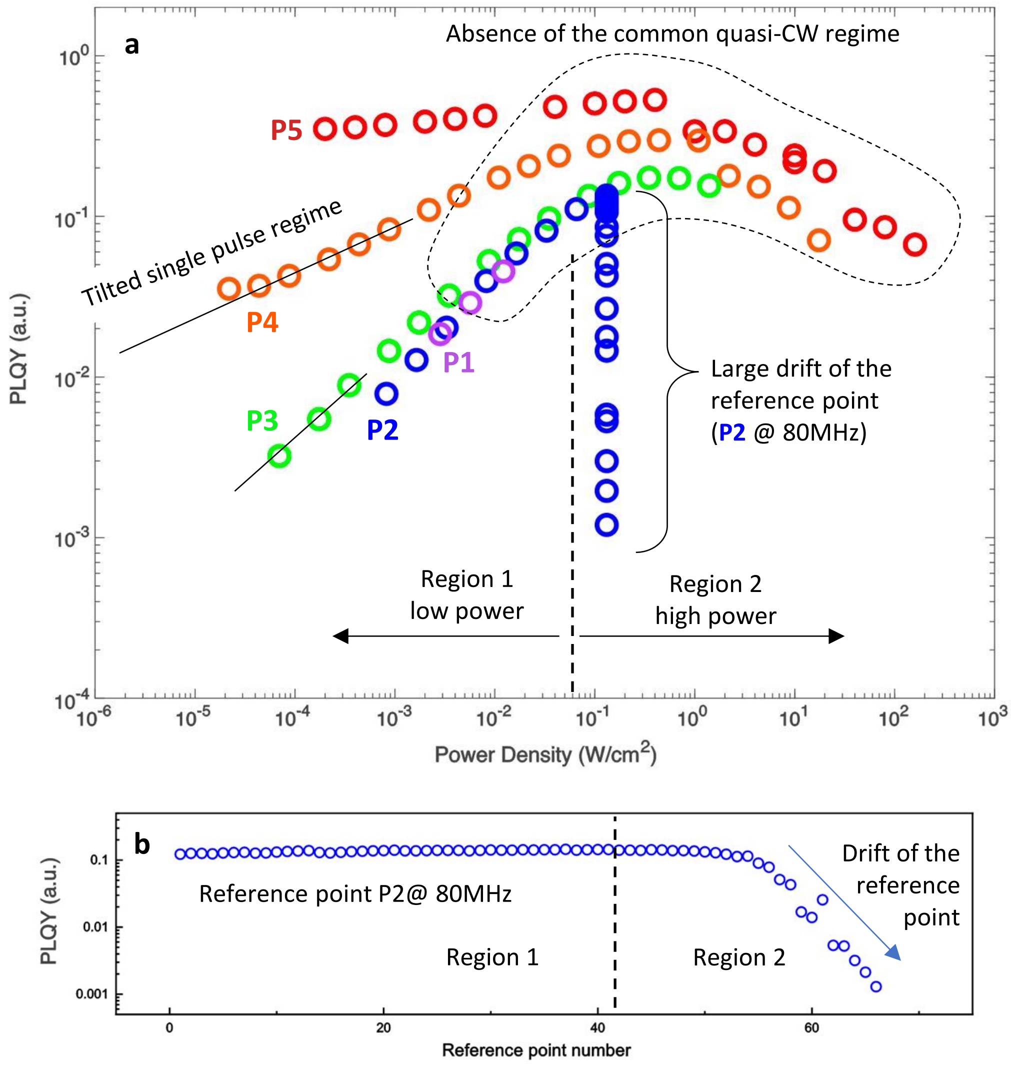

Supplementary Fig. 3. a) Standard[8] measurement of the PLQY(f,P) map for $CsPbBr_3$ polycrystalline film. Two regions are separated: Region 1, where the input power density is low, and Region 2 where the input power density is high (> 0.05 W/cm^2). The map exhibits very strong artifacts due to sample instability (see notes in the plot). b) Evolution of the reference point.

Supplementary Table 2. The conditions for acquiring of the experimental points listed in the measurement order (from 1 to 125) in the experiment shown in **Supplementary Fig.3**. The two regions of low and high intensity are separated. The rows showing the reference points are filled with the white color to separate them from the rows (filled by light green) showing the actual points of the PLQY(f, P) map. The font color highlights the fluences P1-P5 according to the color scheme used in **Supplementary Fig.3a and Supplementary Table 1.**

Region 1

Point number	Excitation Filter 1, Optical Density	Excitation Filter 2, Optical density	Emission Filter, Optical density	Exposure Time (ms)	f (Hz)	Shutter (0 - opened, 1 - closed)
1	3	0	0	100	80000000	0
2	4	0	0	30000	20000000	0
3	3	0	0	100	80000000	0
4	4	0	0	15000	40000000	0
5	3	0	0	100	80000000	0
6	4	0	0	8000	80000000	0
7	3	0	0	100	80000000	0
8	3	0	0	30000	500000	0
9	3	0	0	100	80000000	0
10	3	0	0	20000	1000000	0
11	3	0	0	100	80000000	0
12	3	0	0	10000	2000000	0
13	3	0	0	100	80000000	0
14	2	0	0	100000	4000	0
15	3	0	0	100	80000000	0
16	2	0	0	50000	10000	0
17	3	0	0	100	80000000	0
18	2	0	0	20000	20000	0
19	3	0	0	100	80000000	0
20	2	0	0	20000	50000	0
21	3	0	0	100	80000000	0
22	2	0	0	10000	100000	0
23	3	0	0	100	80000000	0
24	2	0	0	5000	200000	0
25	3	0	0	100	80000000	0
26	1	0	0	100000	100	0
27	3	0	0	100	80000000	0
28	1	0	0	100000	200	0
29	3	0	0	100	80000000	0
30	1	0	0	50000	400	0
31	3	0	0	100	80000000	0
32	1	0	0	20000	1000	0
33	3	0	0	100	80000000	0
34	1	0	0	10000	2000	0
35	3	0	0	100	80000000	0
36	1	0	0	5000	4000	0
37	3	0	0	100	80000000	0
38	1	0	0	2000	10000	0
39	3	0	0	100	80000000	0
40	1	0	0	1000	20000	0
41	3	0	0	100	80000000	0
42	0	0	0	5000	100	0
43	3	0	0	100	80000000	0
44	0	0	0	5000	200	0
45	3	0	0	100	80000000	0
46	0	0	0	2000	400	0
47	3	0	0	100	80000000	0
48	0	0	0	1000	1000	0
49	3	0	0	100	80000000	0
50	0	0	0	500	2000	0
51	3	0	0	100	80000000	0
52	0	0	0	300	4000	0
53	3	0	0	100	80000000	0
54	3	0	0	5000	5000000	0
55	3	0	0	100	80000000	0
56	2	0	0	2000	500000	0
57	3	0	0	100	80000000	0
58	1	0	0	1000	50000	0
59	3	0	0	100	80000000	0
60	3	0	0	2000	10000000	0
61	3	0	0	100	80000000	0
62	2	0	0	1000	1000000	0
63	3	0	0	100	80000000	0
64	1	0	0	500	100000	0
65	3	0	0	100	80000000	0
66	0	0	0	70	10000	0
67	3	0	0	100	80000000	0
67	3	0	0	1000	20000000	0
68	3	0	0	100	80000000	0
68	3	0	0	500	40000000	0
69	3	0	0	100	80000000	0
70	2	0	0	500	2000000	0
71	3	0	0	100	80000000	0
72	1	0	0	500	200000	0
73	3	0	0	100	80000000	0
74	0	0	0	70	20000	0

Region 2

Point number	Excitation Filter 1, Optical Density	Excitation Filter 2, Optical density	Emission Filter, Optical density	Exposure Time (ms)	f (Hz)	Shutter (0 - opened, 1 - closed)
75	3	0	0	100	80000000	0
76	2	0	0	500	5000000	0
77	3	0	0	100	80000000	0
78	1	0	0	200	500000	0
79	3	0	0	100	80000000	0
80	0	0	1	70	50000	0
81	3	0	0	100	80000000	0
82	3	0	0	100	80000000	0
83	3	0	0	100	80000000	0
84	2	0	0	200	10000000	0
85	3	0	0	100	80000000	0
86	1	0	0	100	1000000	0
87	3	0	0	100	80000000	0
88	0	0	1	70	100000	0
89	3	0	0	100	80000000	0
90	2	0	0	100	20000000	0
91	3	0	0	100	80000000	0
92	1	0	1	200	2000000	0
93	3	0	0	100	80000000	0
94	0	0	1	70	200000	0
95	3	0	0	100	80000000	0
96	2	0	1	200	40000000	0
97	3	0	0	100	80000000	0
98	1	0	1	100	5000000	0
99	3	0	0	100	80000000	0
100	0	0	2	100	500000	0
101	3	0	0	100	80000000	0
102	2	0	1	100	80000000	0
103	3	0	0	100	80000000	0
104	1	0	2	500	10000000	0
105	3	0	0	100	80000000	0
106	0	0	2	70	1000000	0
107	3	0	0	100	80000000	0
108	1	0	2	200	20000000	0
109	3	0	0	100	80000000	0
110	0	0	3	200	2000000	0
111	3	0	0	100	80000000	0
112	1	0	2	200	40000000	0
113	3	0	0	100	80000000	0
114	0	0	3	100	5000000	0
115	3	0	0	100	80000000	0
116	1	0	2	100	80000000	0
117	3	0	0	100	80000000	0
118	0	0	3	100	10000000	0
119	3	0	0	100	80000000	0
120	0	0	4	200	20000000	0
121	3	0	0	100	80000000	0
122	0	0	4	200	40000000	0
123	3	0	0	100	80000000	0
124	0	0	4	70	80000000	0
125	3	0	0	100	80000000	0

Supplementary Table 3. The experimental points located in the order of their acquisition (from 1 to 42) in the experiment shown in **Supplementary Fig. 4a, b**. The two regions of low and high intensity are separated. The rows showing the reference points are filled with white color to separate them from the rows (filled with light green) showing the actual points of the PLQY(f, P) map. The font color highlights the fluences P1-P5 according to the standard color scheme used in **Supplementary Fig. 4a, b and Supplementary Table 1.**

Point number	Excitation Filter 1, Optical Density	Excitation Filter 2, Optical density	Emission Filter, Optical density	Exposure Time (ms)	f (Hz)	Shutter (0 - opened, 1 - closed)
1	4	0	0	40000	1000000	0
2	4	0	0	8000	5000000	0
3	4	0	0	2000	10000000	0
4	4	0	0	1000	20000000	0
5	4	0	0	500	40000000	0
6	4	0	0	250	80000000	0
7	3	0	0	180000	10000	0
8	3	0	0	60000	30000	0
9	3	0	0	20000	100000	0
10	3	0	0	4000	500000	0
11	3	0	0	1000	2000000	0
12	3	0	0	250	5000000	0
13	3	0	0	140	10000000	0
14	3	0	0	70	20000000	0
15	3	0	0	70	40000000	0
16	3	0	0	70	80000000	0
17	2	0	0	540000	300	0
18	2	0	0	180000	1000	0
19	2	0	0	60000	3000	0
20	2	0	0	15000	10000	0
21	2	0	0	4000	30000	0
22	2	0	0	2000	100000	0
23	1	0	0	240000	10	0
24	1	0	0	80000	30	0
25	1	0	0	40000	100	0
26	1	0	0	20000	300	0
27	1	0	0	5000	1000	0
28	1	0	0	2000	3000	0
29	1	0	0	1000	10000	0
30	0	0	0	5000	100	0
31	0	0	0	3000	300	0
32	0	0	0	1000	1000	0
33	0	0	0	300	3000	0
34	0	0	0	100	10000	0
35	2	0	0	31,25	40000000	0
36	2	0	0	15,63	80000000	0
37	1	0	0	2,50	40000000	0
38	1	0	0	1,25	80000000	0
39	0	0	0	0,50	20000000	0
40	0	0	0	0,25	40000000	0
41	0	0	0	0,13	80000000	0
42	4	0	0	250	80000000	0

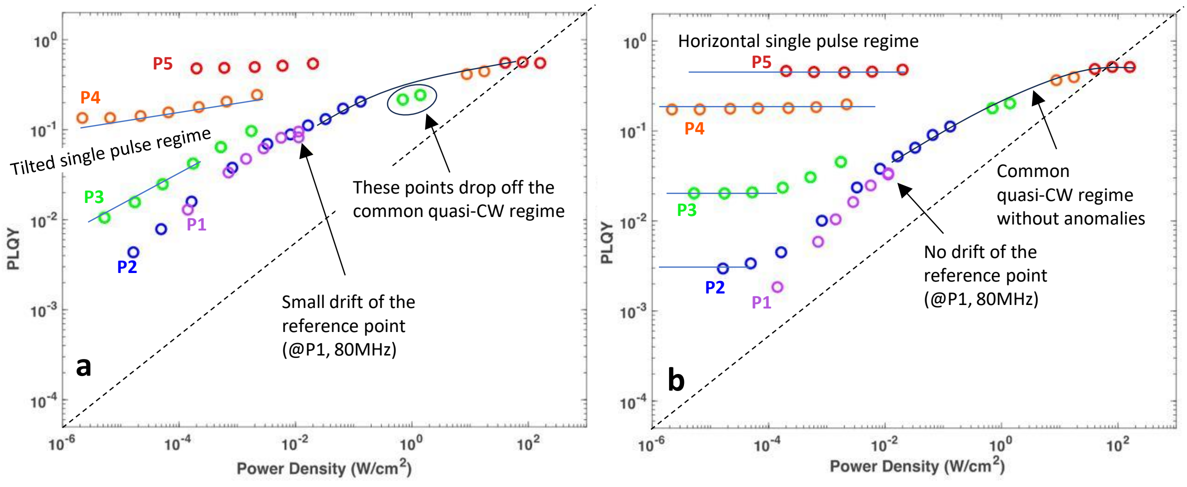

Supplementary Fig. 4. The PLQY(f,P) plots with the optimized exposure time according to **Supplementary Table 3.** a) The PLQY(f,P) plot for the initial state of the sample (as-prepared), there are some features revealing sample instability (see notes in the plot). b) PLQY(f,P) map for the sample pre-exposed for 20 seconds at P5 fluence at 80 MHz frequency. By this procedure we prepared $\vec{S}_1$ state of the sample, which was stable enough to measure a reliable PLQY(f,P) map which was used for modelling, see notes on the figures for details.

Due to technical limitations of the mechanical shutter used in the setup, the minimum exposure time in the standard measurement protocol was 70 ms, which was obviously too long, leading to changes of the sample state when excited at high power densities (Region 2). Importantly, from the point of view of the signal strength, a much shorter exposure time would be sufficient to record the PL signal at the high-power density region, for example, 70 ms could be replaced by 1 ms if the shutter would allow it.

Therefore, in order to improve the sample state stability (i.e. to reach a condition which is as close as possible to $\frac{d\overrightarrow{S(f,P)}}{dt} = 0$), the dose of light irradiating the sample was minimized by changing the manner in which the exposure time was set. Instead of using a mechanical shutter, the laser itself was used to determine the irradiation time of the sample. The laser was run in a pulse-burst mode with a very large time interval (1 s) between the bursts (enabled by using a Sepia 828 PicoQuant controller). The mechanical shutter was then synchronized with the laser to open in such a way that within its open time only one pulse burst was able to reach the sample per one measured data point. This approach made it possible to set the exposure for all the data points to the minimal value determined by the signal to noise ratio. For example, the exposure time of the sample to the highest power density 160 W/cm^2 (P5, 80MHz,) was decreased more than a factor of 500 (from 70 to 0.13 ms). This greatly increased the stability of the sample. However, we note that measurements at some combinations of f and P belonging to Region 2 were avoided since even the minimal possible exposure led to changes in the sample. The protocol for this experiment is provided in **Supplementary Table 3**.

A PLQY (f,P) map measured according to **Supplementary Table 3** is shown in **Supplementary Fig.4a.** The difference between the 1st and last values of the measured reference point (P1, 80 MHz) decreases significantly. Still, the sample demonstrated some features incompatible with SRH+ model (see notes on the **Supplementary Fig.4a**). We conclude that the initial, as-prepared state of the sample $\vec{S}_0$, is extremely sensitive to light and

is prone to easily change into another state $\vec{S}_i$ during the measurements even when taking significant precautions.

It was found that by exposing the sample to the 160 W/cm^2 for 20 s, it was possible to induce a state (designated as $\vec{S}_1$) which is much more stable as the initial state of the sample. Several experiments shown in the main text (**Fig. 2a, 3d, 3e**) were carried out for samples prepared in this way. The PLQY(f,P) plot corresponding to this state is shown in **Supplementary Fig.4b.** In such a state, the reference point was stable and the PLQY(f,P) map did not exhibit any features that could not be well described by the SRH+ model. This data was utilized for modelling using the SRH+ model to extract the model parameters (see **Supplementary Note 5.6** and **Supplementary Fig.10**). Note, however, that the parameters extracted from this data cannot be completely assigned to the $\vec{S}_1$ state. The PLQY(f,P) mapping experiment lasts about 1 h and some relaxation processes of the sample state during this time cannot be excluded.

Supplementary Note 4. Paired Pulse Facilitation of $CsPbBr_3$ and $MAPbI_3$ Memlumors

Here the synaptic plasticity for perovskite memlumors is demonstrated. By analogy to memristors, in which the synaptic weight is the conductance, in the memlumor case, the synaptic weight is the PLQY. The relative PLQY for each pulse can be obtained by dividing the PL output by the input light intensity. Thus, the difference between the PL of two nearby PL responses excited by the same input pulse intensity is the difference in the synaptic weight of a memlumor. By varying the delay time between the input pulses from the 12.5 ns to the 2.5 μs (**Supplementary Fig.5a)**, it is possible to demonstrate the typical paired-pulse facilitation behavior for the $CsPbBr_3$ film (**Supplementary Fig.5b)** and the $MAPbI_3$ film (**Supplementary Fig.5c)** memlumors.

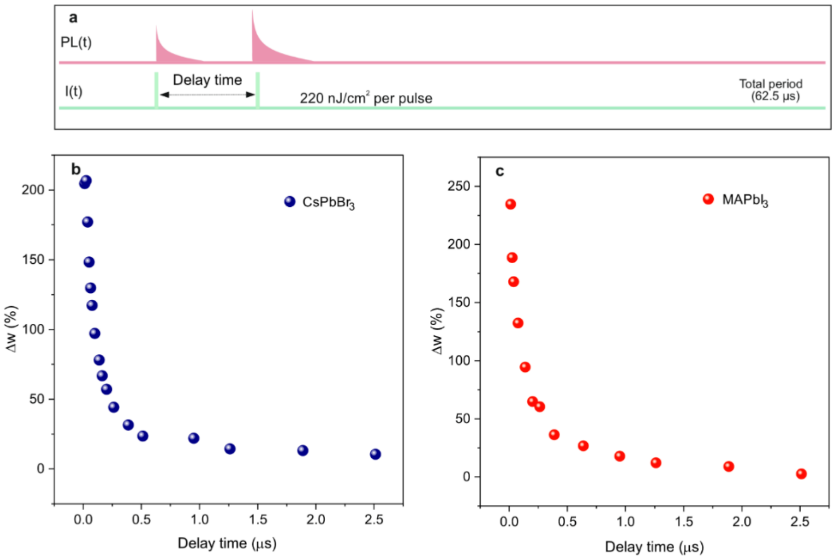

Supplementary Fig. 5. Measurement of the paired pulse facilitation (PPF). a) Typical experiment to detect PPF. When the delay time between the two nearby response pulses changes, the relative intensity of the responses also changes. b) PPF for $CsPbBr_3$ and a) for $MAPbI_3$ film memlumors excited at the P3 fluence.

Supplementary Note 5. Theoretical Modeling and Calculations

S5.1. Shockley-Read-Hall (SRH) model with added radiative recombination (SRH+radiative)

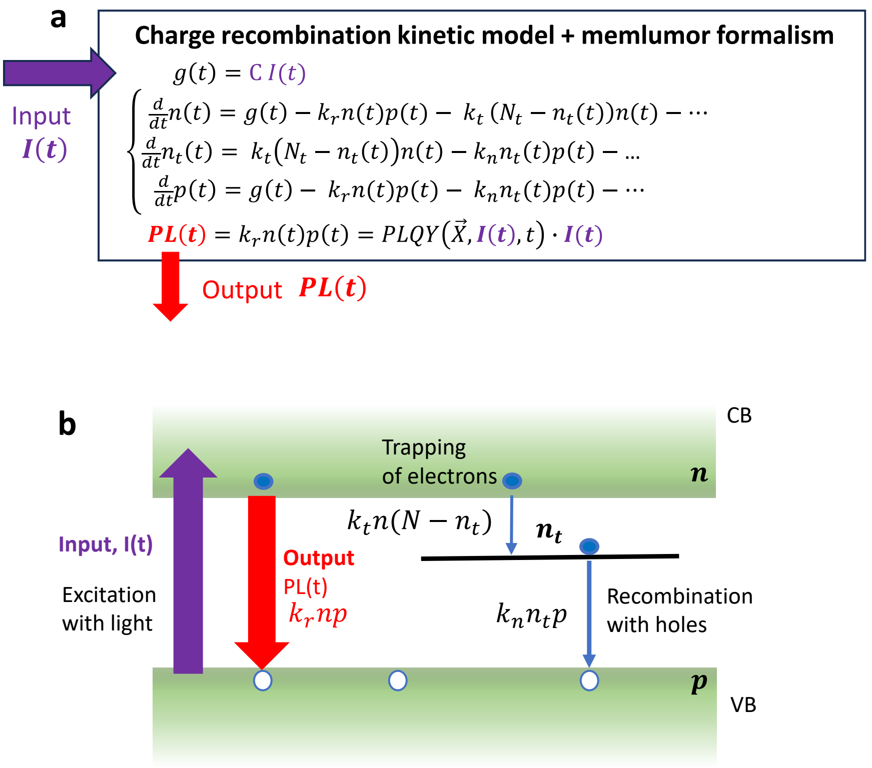

Supplementary Fig. 6. (a) Illustration of the origin of the state-dependent PLQY of a luminescent semiconductor with charge trapping. (b) SRH charge recombination model with added radiative recombination (SRH+radiative). In this example the defect states are electron traps.

The SRH model considers carrier recombination occurring via trapping states located within the band gap.[13] A SRH model with added radiative term (SRH + radiative) can be used to explain the charge carrier recombination in a luminescent semiconductor.[7] The processes considered in the SRH model are illustrated in **Supplementary Fig.6b** where an excess of charge carriers is generated by photoexcitation. These charge carriers can either recombine non-radiatively via the trap states (electron trapping followed by recombination with a free hole) or radiatively leading to photoluminescence (PL) that can be detected experimentally. Mathematically, these processes can be described by a system of three differential equations. These equations describe the density of electrons in the conduction band (n), the density of holes in the valence band (p) and the density of trapped electrons (n_t) at the trap level:

$$\frac{d}{dt}n(t) = g(t) - k_r np - k_t(N_t - n_t)n \qquad \text{S5.1}$$

$$\frac{d}{dt}n_t(t) = k_t(N_t - n_t)n - k_n n_t p \qquad \text{S5.2}$$

$$\frac{d}{dt}p(t) = g(t) - k_r np - k_n n_t p \qquad \text{S5.3}$$

where, g(t) is the density of charge carriers generated by the excitation light per second, k_r is the radiative recombination rate constant, k_t is the electron trapping rate constant, N_t is the electron trap density and, k_n is the non-radiative recombination rate constant. The traps are considered to be deep enough to make de-trapping of electrons negligible.

The generation rate g(t) [excitations/(cm^3s)] is a function of the excitation intensity I(t) [W/cm^2]. In the simplest case considered here[7] we assume that the sample is rather thin and the charge carriers redistribute very quickly after their generation over the thickness d of the sample. In this case $g(t) = Abs\, I(t)/(d\, h\nu) = C\, I(t)$, where d – sample thickness and $h\nu$ – excitation photon energy, and Abs - absorption coefficient of the sample (see **Supplementary Note 2.2**) and $C = Abs/(dh\nu)$ (**Supplementary Fig. 6a**).

Since an intrinsic semiconductor is charge neutral, the following condition takes place:

$$n(t) + n_t(t) = p(t) \qquad \text{S5.4}$$

In the presence of chemical doping the condition for charge neutrality becomes

$$n(t) + n_t(t) + cd = p(t)$$

Where cd is the doping density, cd should be negative in the case of n-doping or positive in the case of p-doping.

The PL intensity in this model is given by

$$PL(t) = k_r n(t) p(t) \qquad \text{S5.5}$$

Trapping of electrons by the trap states leads to an excess of holes in the valence band and results in the effect commonly referred to as photodoping.

In the equations above electron trapping was assumed. However, the same equations also apply for hole trapping if one replaces n with p and *vice versa*. Consequently, neither the model nor this type of experiment make it possible to distinguish between photodoping induced by electron trapping or by hole trapping.

5.2. Extended Shockley-Read-Hall model (SRH+) with added Auger processes.

The model described in the section above is valid at low excitation conditions only, such that the third order Auger-assisted processes can be neglected. To explain the carrier recombination at high excitation conditions, the so-called SRH+ model[7] is applied. This model includes Auger recombination and Auger-assisted charge trapping (**Supplementary Fig.7**).

The kinetic equations of the SRH+ model can be written as follows:[7]

$$\frac{d}{dt}n(t) = g(t) - k_r np - k_t(N_t - n_t)n - k_e np(N_t - n_t) - k_a np^2 \qquad \text{S5.6}$$

$$\frac{d}{dt}n_t(t) = k_t(N_t - n_t)n + k_e np(N_t - n_t) - k_n n_t p \qquad \text{S5.7}$$

$$\frac{d}{dt}p(t) = g(t) - k_r np - k_n n_t p - k_a np^2 \qquad \text{S5.8}$$

along with the condition of charge neutrality,

$$n(t) + n_t(t) = p(t)$$

Here, k_e, and k_a are the Auger-assisted electron trapping rate constant and, Auger-assisted recombination coefficient, respectively.

In this work, the SRH+ model was used to fit the PLQY (f, P) data and the PL decays (**Supplementary Fig.10)**. These data sets were obtained for a very broad range of excitation conditions making it necessary to consider the third order processes. Modeling of this experimental data made it possible to obtain all the parameters of the model (all rates and trap concentration) (**Supplementary Table 4**).

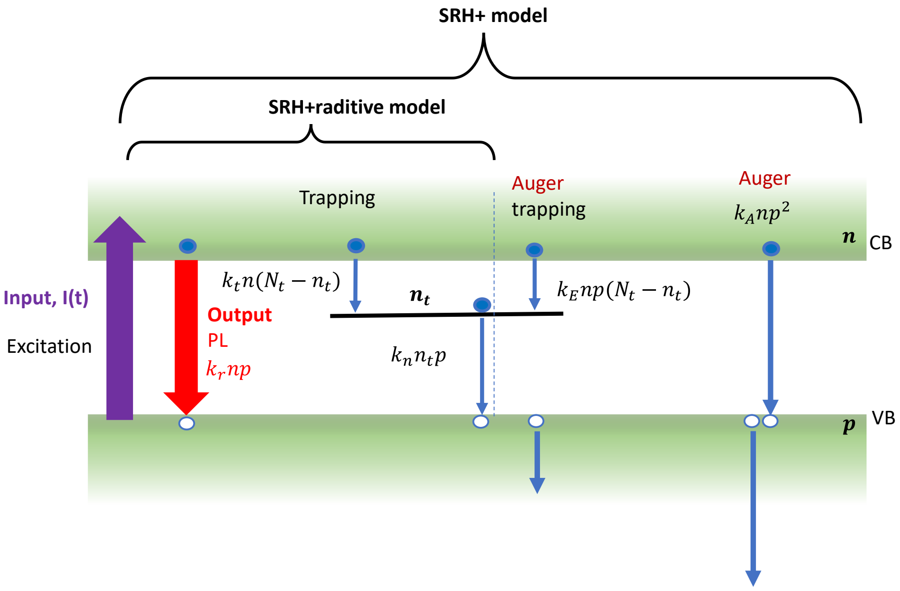

Supplementary Fig. 7. Schematic diagram of the processes considered in the SRH+ model, from left to right: photoexcitation, radiative recombination, electron trapping, recombination of the trapped electron with a hole, Auger-assisted electron trapping (in the presence of an empty trap and a hole) and Auger-assisted non-radiative recombination (electron recombines with a hole in the presence of another hole).[8]

5.3. Pulsed photoexcitation in the framework of the SRH+ model

In a typical experiment, the PL is excited by a very short laser pulse at time t=0. Assuming that the pulse width is much shorter than all relaxation times in the system, the generation g(t) can be approximated as a δ-function centered around t=0 creating the initial concentration of electron-hole pairs n_0. However, it is necessary to consider that practically no experiments are carried out with just one excitation pulse, instead the excitation pulse is repeated with a certain repetition period T. So, it is important to consider that the excitations created by the previous pulse may not have yet decayed by the moment of the next pulse. Under such circumstances, the charge carrier kinetic equations can be expressed as follows (see more details in SI to ref[7]):

$$\frac{d}{dt}n(t) = -k_r np - k_t(N_t - n_t)n - k_e np(N_t - n_t) - k_a np^2 \qquad \text{S5.9}$$
$$\frac{d}{dt}n_t(t) = k_t(N_t - n_t)n + k_e np(N_t - n_t) - k_n n_t p \qquad \text{S5.10}$$
$$\frac{d}{dt}p(t) = -k_r np - k_n n_t p - k_a np^2 \qquad \text{S5.11}$$

with the periodic boundary conditions:

$$n(0) = n(T) + n_0$$
$$p(0) = p(T) + n_0$$
$$n_t(0) = n_t(T)$$

and the condition of charge neutrality

$$n(t) + n_t(t) = p(t)$$

Equations S5.9 to S5.11 can be numerically solved using given initial and periodic boundary conditions to obtain the concentration of electrons and holes as a function of time. Subsequently, the value of PL quantum yield (PLQY which we measured experimentally) in a periodic pulsed excitation experiment can be calculated as:

$$PLQY = \frac{Number\ of\ photons\ emitted\ per\ one\ pulse}{number\ of\ e\text{–}h\ pairs\ created\ by\ one\ pulse} = \frac{k_r}{n_0}\int_{t'}^{t'+T} n(t)p(t)dt \qquad \text{S5.12}$$

where t' is the starting time of the integration over the pulse repetition period. Note that, this starting time t' must be chosen with care as will be discussed below.

The distance between excitation pulses (the excitation period T=1/f, where f is the pulse repetition rate) in the experiments can be as short as 12.5 ns (corresponding to 80 MHz repetition rate) or as long as hundreds of microseconds. If the repetition period of the excitation is so long that the densities of excess electrons, trapped electrons and excess holes decay to values very close to zero, this is called the *single-pulse excitation regime*. In this regime neither a small decrease nor an increase of the excitation period changes the PL response to a single excitation pulse.

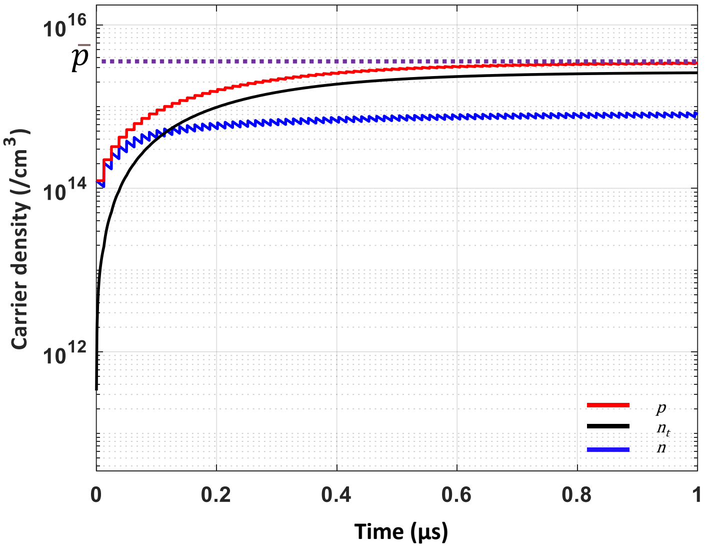

Supplementary Fig. 8 Evolution of the electron density in conduction band (n), the hole density in valence band (p), and the trapped electron density (n_t) under continuously applied pulsed excitation with repetition rate 80 MHz and excitation power P2 (see **Supplementary Table 1**) which starts at time zero. Note the increase and the eventual saturation of $p \approx n_t = \bar{p}$. In this example the equilibrated concentration of free electrons is about 3 times less than the concentration of the free holes due to the photodoping effect. The time needed [illegible]

When the period becomes shorter, some excited species remain in the system when the next pulse arrives. Therefore, the PL dynamics starts to depend on the pulse period T. In the limiting case of a short distance between pulses, PLQY of the semiconductor starts to depend only on

the time-averaged power density (W/cm^2), which is proportional to the product f×P. This regime is called *quasi-CW excitation regime*.[7]

When the SRH+ model is used to calculate the parameters which can be compared with experimental data, *e.g.* with PL(t) measured by the TCSPC method, it is important to realize that such experimental data are measured in the quasi-steady state condition in the sense that the sample is repeatedly excited with laser pulses. Because the accumulation time of the PL decay (tens of seconds) is much larger than the time required for PL to reach the quasi equilibrium, the TCSPC measures an equilibrated response of the system to the pulsed excitation.

If the excitation is the *single-pulse regime*, this equilibrated response is the same as the response to one single pulse. In other cases, one needs to apply multiple pulses until the system comes to an equilibrium. Experimentally, this is always the case because the accumulation time for PL decays is usually tens of seconds which is several orders of magnitude longer than the time required to reach the equilibrium **(Supplementary Fig. 8**). To reproduce the experimental conditions in the calculations, it is necessary to sequentially calculate as many repeated pulses as needed to establish the quasi-steady state values of the electron and hole densities.

This is illustrated in **Supplementary Fig. 8** where one can see that the time-averaged concentrations of electrons (n) trapped electrons (n_t) and free holes (p) are increasing steadily after each excitation pulse until they reach certain equilibrated values. At this quasi-equilibrium the concentrations still go up and down in response to the laser pulses, however, these dependencies are stable and fully periodic. When the model reaches this quasi-equilibrium condition, PLQY and PL decays are calculated and used to fit the PLQY(f,P) map (see **Supplementary Note 5.5 and 5.6**) and the PL lifetime data.

Taking this into consideration, we must conclude that the time t' in equation S5.12 (the time from which the integration commences) should be larger than the time required for the system to reach the quasi-steady state condition. For calculations of the PL response to bursts of excitation pulses, the bursts were repeated several times with the needed repetition period until the quasi-steady state response is reached.

5.4 Solution of the model equations of SRH+ radiative model in the special case of low excitation fluence

An analytical solution of the equations of the SRH+ radiative model is possible at the limiting case of very low excitation density. In this case, the radiative recombination rate becomes negligible compared to the charge trapping rate. In addition, it is assumed that n_t is significantly smaller than the trap density N_t ($k_r p \ll k_t N_t$ *and* $n_t \ll N_t$). In this case, equations S5.1 – S5.3 can be approximated as:

$$\frac{d}{dt}n(t) = -k_t N_t n \quad \text{S5.13}$$

$$\frac{d}{dt}n_t(t) = k_t N_t n - k_n n_t p \quad \text{S5.14}$$

$$\frac{d}{dt}p(t) = -k_n n_t p \quad \text{S5.15}$$

Then, the periodic solution of S5.13 can be given as:

$n(t) = \frac{n_0 \exp(-k_t N_t t)}{1-\exp(-k_t N_t T)}$ S5.16

Single pulse regime under low excitation condition:

If the period T is sufficiently large to satisfy the following conditions:

$k_t N_t T \gg 1 \; and \; k_n n_0 T \gg 1$,

the system is in the *single-pulse excitation regime*.

From equation S5.16 it follows that:

$$n(t) = n_0 \exp(-k_t N_t t)$$

This indicates that the electron density in the conduction band drops exponentially with the characteristic time $1/k_t N_t$. It can be seen from equation S5.13- S5.15 that the concentration of holes remains almost constant over the time it takes n(t) to fall by a factor of e, provided that

$$t_d k_n n_0 = \frac{k_n n_0}{k_t N_t} \ll 1$$

In this case, one can substitute

$$p(t) = n_0$$

Thus, from equation S5.5 it follows that:

$PL(t) = k_r n_0^2 \exp(-k_t N_t t)$ S5.17

and, $PLQY = \frac{k_r}{k_t N_t} n_0$ S5.18

5.5 Modelling protocol for the extraction of the SRH+ model parameters

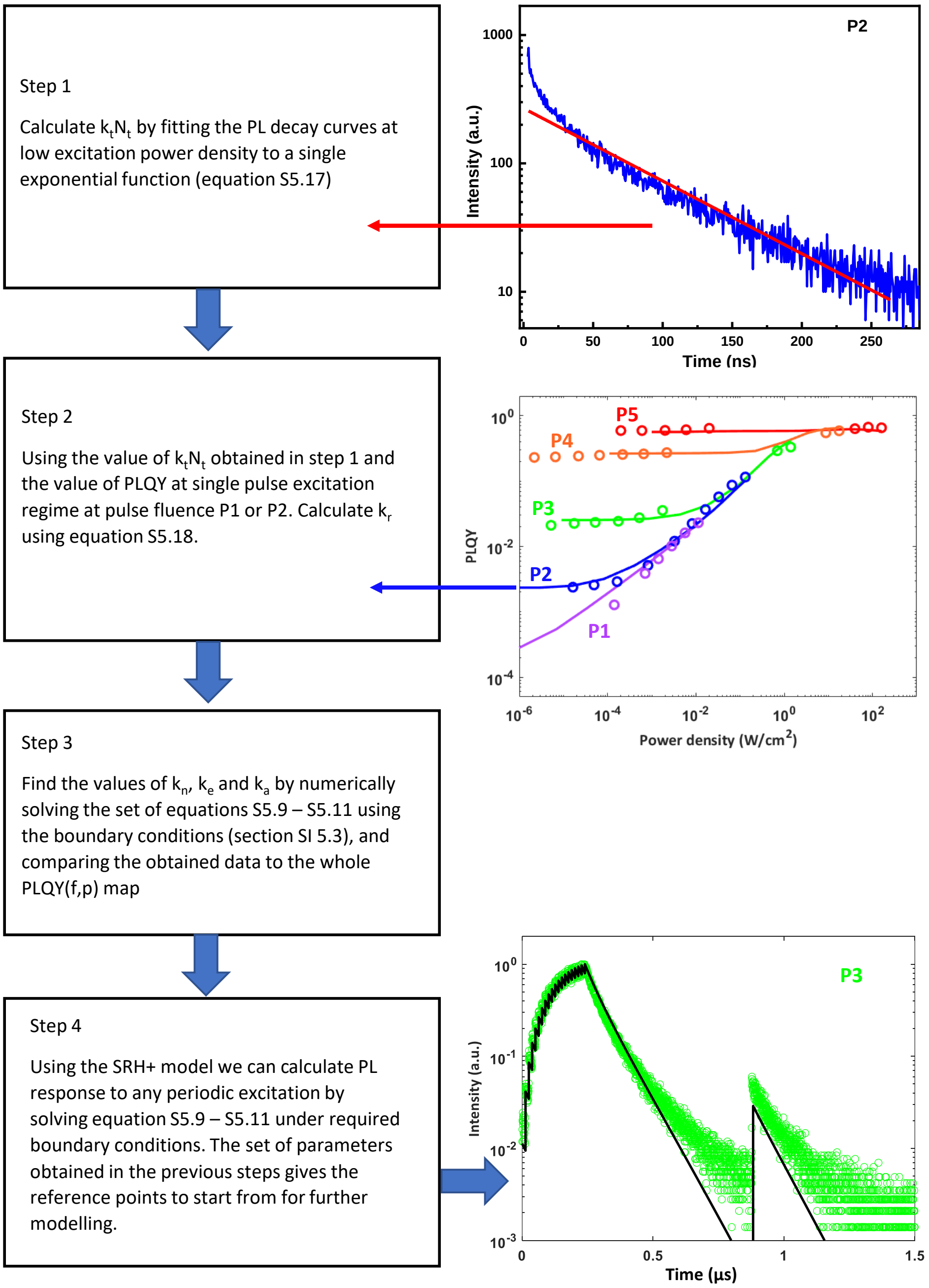

Supplementary Fig. 9. Block diagram of the fitting procedure. The step 4 is illustrated by an example of a modelled experimental PL response to a write/read cycle, where the write is a burst of 20 pulses at P3 fluence and the read is a single pulse at the same fluence with a delay time of $\approx 0.6\ \mu s$ after the write (green – experiment, black – modelling). The parameters for the modelling were those obtained in Step 3 (**Supplementary Table 4**).

5.6. Extracting parameters according to the modeling protocol. Fitting experimental data for the $CsPbBr_3$ film memlumors.

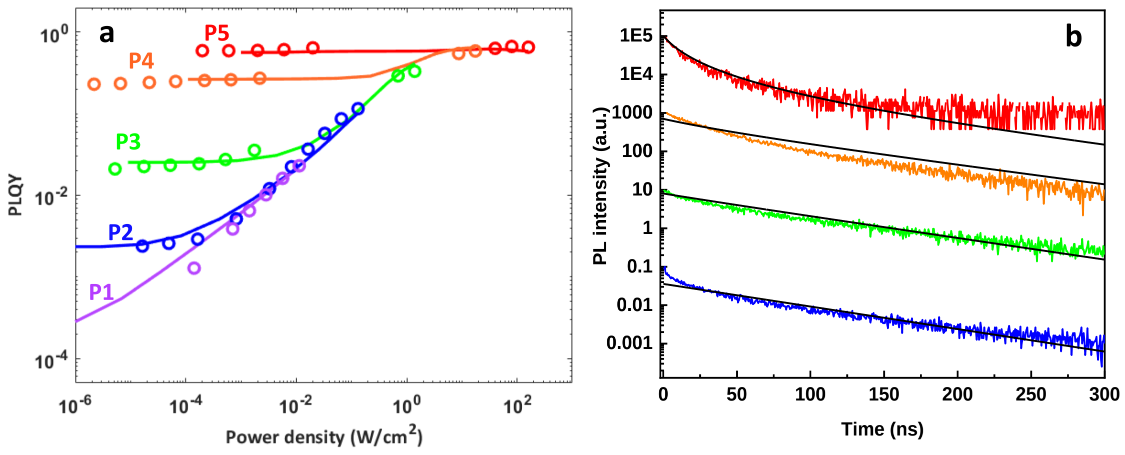

Supplementary Fig. 10. PLQY(f,P) map and the time-resolved PL for the $CsPbBr_3$ film and theoretical modelling of these data by the SRH+ model. a) The full PLQY(f,P) map. b) Time-resolved PL for different pulse fluences P1-P5 measured at 16 kHz repetition rate. The measurement of the PLQY(f,P) map is described in **Supplementary Note 2.4 and 3.**

Supplementary Table 4. The parameters of the SRH+ model for the $CsPbBr_3$ film memlumor obtained from the PLQY (f,P) mapping and PL decays (protocol **in Supplementary Note 5.5**). These parameters approximately correspond to the $\vec{S}_1$ state of the $CsPbBr_3$ film (marked by *). These parameters used in **Fig. 2a** and **Fig. 2b** of the main text.

$k_{t1}^{*}N_{t1}^{*}$	**$1.35\text{x}10^{7}$ s^{-1}**
N_{t1}^{*}	**$7.85\text{x}10^{15}$ cm^{-3}**
k_{n1}^{*}	**$7.93\text{x}10^{-10}$ $cm^{3}s^{-1}$**
k_r	**$2.56\text{x}10^{-10}$ $cm^{3}s^{-1}$**
k_e	**$2.10\text{x}10^{-28}$ $cm^{6}s^{-1}$**
k_a	**$1.40\text{x}10^{-27}$ $cm^{6}s^{-1}$**

Supplementary Table 5. The parameters used to fit the experimental PL response of the $\vec{S}_1$ state of the $CsPbBr_3$ film memlumor in **Fig.3e** from the main text.

$k_{t1}N_{t1}$	**$1.35\text{x}10^{7}$ s^{-1}**
N_{t1}	**$7.85\text{x}10^{15}$ cm^{-3}**
k_{n1}	**$1.98\text{x}10^{-9}$ $cm^{3}s^{-1}$**
k_r	**$2.56\text{x}10^{-10}$ $cm^{3}s^{-1}$**
k_e	**$2.10\text{x}10^{-28}$ $cm^{6}s^{-1}$**
k_a	**$1.40\text{x}10^{-27}$ $cm^{6}s^{-1}$**

Supplementary Table 6. The parameters used to fit the experimental PL response of the $\vec{S}_0$ state of the $CsPbBr_3$ film memlumor in **Fig. 3e** from the main text. For the modelling of the PL response in the state $\vec{S}_0$ we change only parameters k_t , k_n and N_t (components of the state vector $\vec{S}$) which are the properties of the traps, the other parameters here are the same as for the state $\vec{S}_1$ **in Supplementary Table 5.**

$k_{t0}N_{t0}$	**$2.43x10^7$ s^{-1}**
N_{t0}	**$7.85x10^{15}$ cm^{-3}**
k_{n0}	**$1.19x10^{-8}$ cm^{3}s^{-1}**
k_r	**$2.56x10^{-10}$ cm^{3}s^{-1}**
k_e	**$2.10x10^{-28}$ cm^{6}s^{-1}**
k_a	**$1.40x10^{-27}$ cm^{6}s^{-1}**

5.7 Modelling of the short-term memory effect and its dependence on the parameters of the trap states

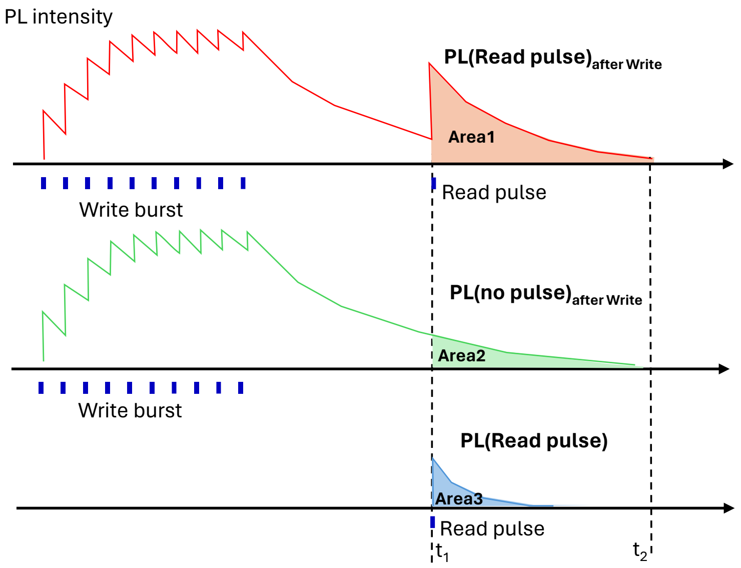

$$M = \frac{\int_{t_1}^{t_2} PL(Read\ pulse)_{after\ Write} dt - \int_{t_1}^{t_2} PL(no\ pulse)_{after\ Write} dt}{\int_{t_1}^{t_2} PL(Read\ pulse) dt} = \frac{Area1 - Area2}{Area3}$$

Supplementary Fig. 11. Explanation of the memory strength parameter M. The write pulse burst creates the memory, which is read by the Read pulse coming at time t_1 (red curve). To evaluate the response to the Read pulse correctly we need to subtract from the signal the “background” PL signal (excited by the Write burst) which can be still present in the region (t_1,t_2) over which the PL signal is integrated. This background signal is measured in the experiment without the Read pulse (green curve). Then the resulted difference is normalized to the refence PL decay integrated over the same interval (blue curve).

This note demonstrates how the capacity of a system to hold memory can be tuned by changing the vector $\vec{S} = (k_t, k_n, N_t)$, and pulse energy P (**Supplementary Fig.11-14**).

The memlumor response is probed by the single pulse coming after the time delay Δt. The memory effect is characterized by the memory strength M explained in **Supplementary Fig.11.** The larger the memory strength, the more information can be stored in the system.

Supplementary Fig.12 shows that among N_t, k_t and k_n parameters the largest effect on M is due to k_n. Decreasing of k_n makes the trapped electrons live longer and thus increasing M (25 times decrease k_n increases M by 10 times). Changing of the trapping rate k_t does not influence M almost at all. Dependence on N_t is also rather weak. Changing of N_t by 25 times leads to only 2 times change in M. Dependence of M on N_t over a broader range is shown in **Supplementary Fig. 13.**

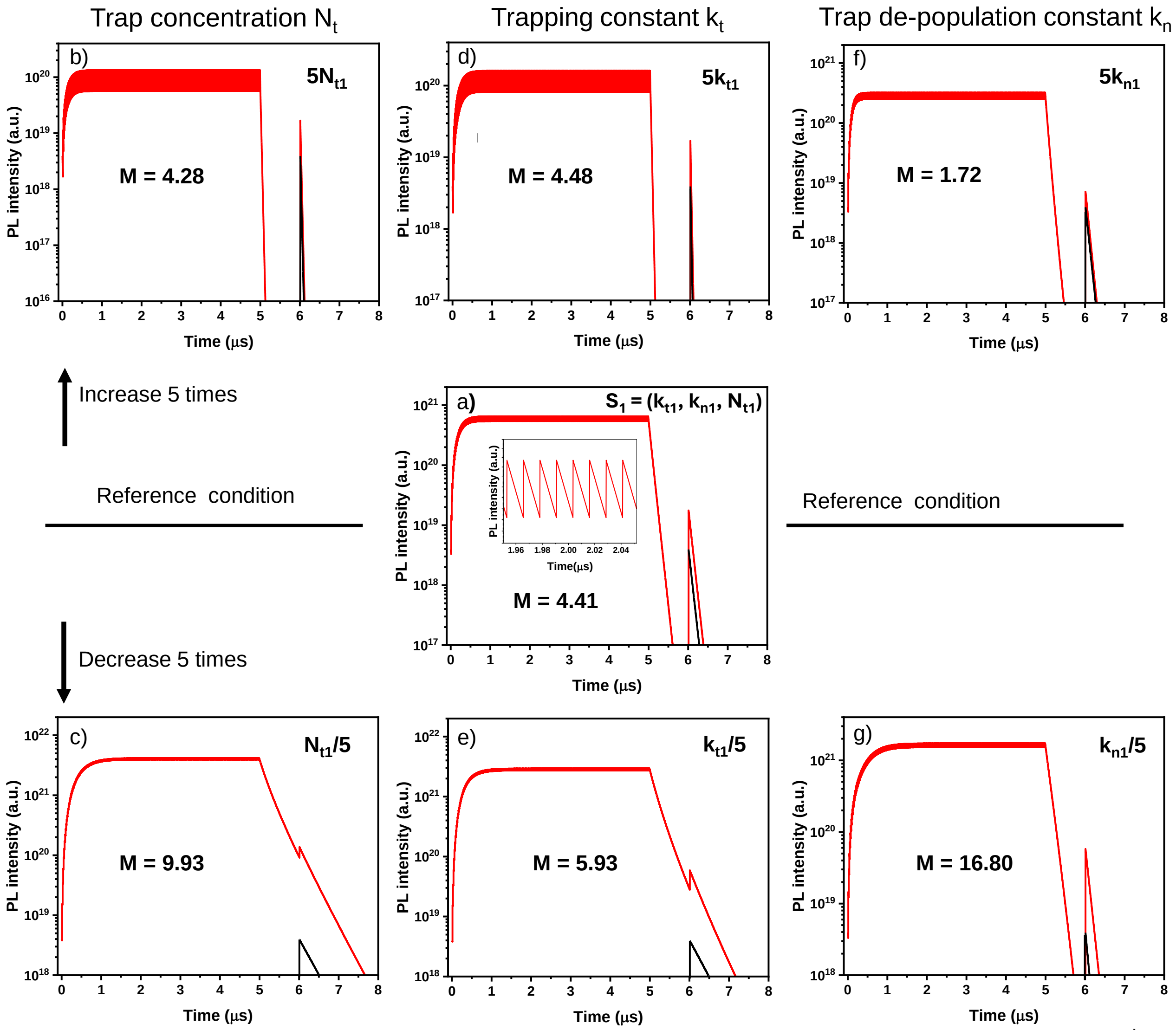

Supplementary Fig. 12. Memory strength (M) dependence on the state vector $\vec{S}_1$ parameters. 400 pulses are used as a Write burst with 80 MHz internal repetition rate (12.5 ns between pulses). The Read pulse comes after 1 microsecond delay. The signal integration time t_1 (see Supplementary Fig.11) is equal to the arrival time of the Read pulse, while the length of integration t_2-t_1 equals to 10 microseconds. For all panels the pulse fluence P2 is used (see **Supplementary Table 1**). Panels a-g show the PL response calculated for different state vector $\vec{S}$. a) $\vec{S} = \vec{S}_1 = (k_{t1}, k_{n1}, N_{t1})$ (**Supplementary Table 5**), this is the condition of our experiments. The other panels show the situation where one of the parameters deviates from the reference condition, namely: b) $N_t = N_{t1}/5$, c) $N_t = 5N_{t1}$, d) $k_t = k_{t1}/5$, e) $k_t = 5k_{t1}$, f) $k_N = k_{N1}/5$ and g) $k_N = 5k_{N1}$.

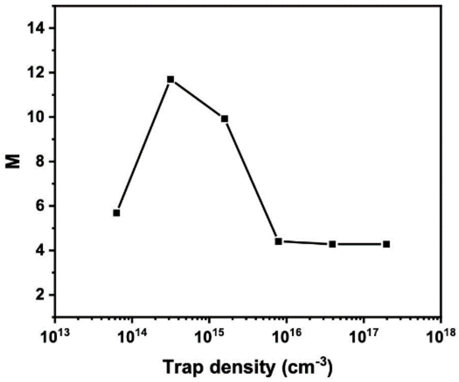

Supplementary Fig. 13. Calculated memory strength (M) dependence on the trap density N_t, the other parameters correspond to the vector $\vec{S}_1$. The experiment is the same as described in Supplementary Fig.12.

Dependence on the pulse fluence (**Supplementary Fig. 14**) is rather strong. It shows that decreasing the pulse fluence increases M which ranges 28 for P1 and 1.02 for P4. This dependence is expected because at high excitation fluence the radiative and Auger processes dominate over the trap-mediated dynamics responsible for the memory effect.

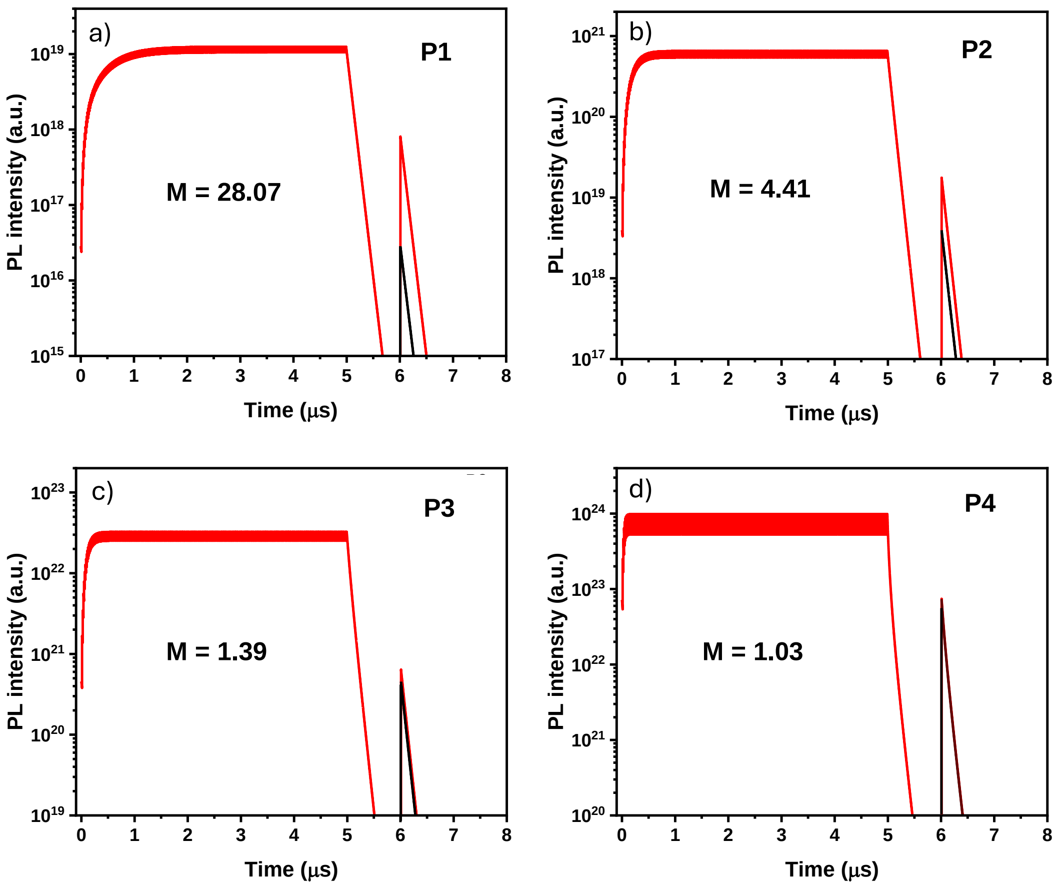

Supplementary Fig. 14. a-d) The memory strength M is calculated for the pulse fluences P1, P2, P3 and P4, respectively (see **Supplementary Table 1** for the fluence values). 400 pulses are used as a Write burst with 80 MHz internal repetition rate (12.5 ns between pulses). The conditions for the analysis are the same as Supplementary Fig.12. All modeling parameters correspond to $\vec{S}_1$ state of the $CsPbBr_3$ memlumor (parameters from **Supplementary Table 5**). The higher the pulse fluence the smaller the memory strength.

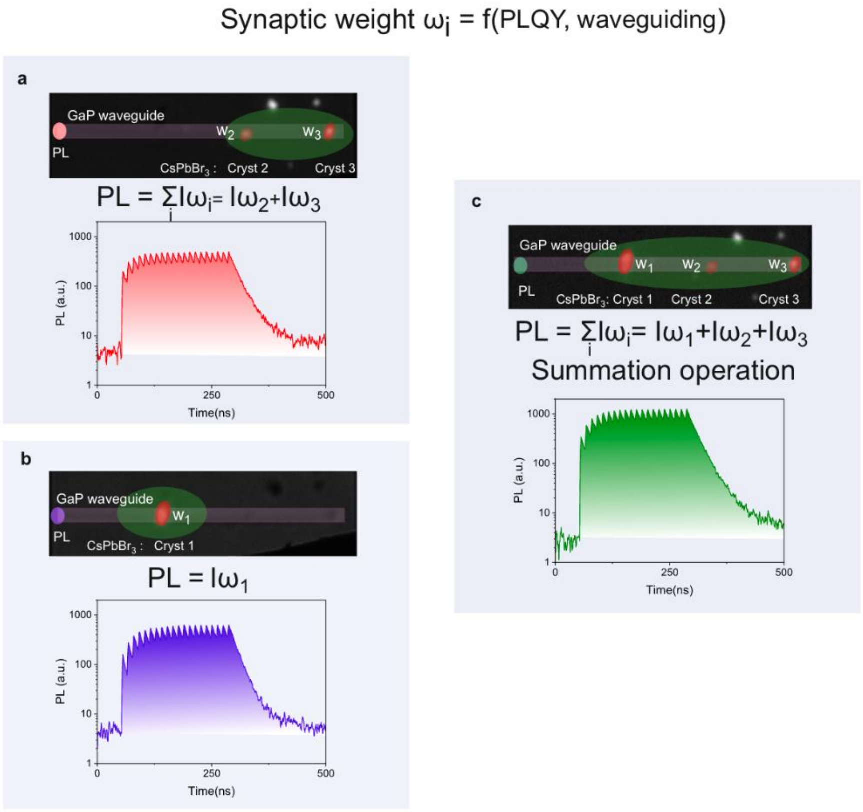

Supplementary Fig. 15. Experiment with a GaP waveguide. Three $CsPbBr_3$ crystals are attached to the GaP waveguide. Their PL propagates in the waveguide to be collected from its edge. The light spot (green) can be controlled so it can illuminate 1, 2 or 3 crystals (panels a, b and c, respectively).

Supplementary Note 6. $CsPbBr_3$ Memlumor Crystals Integrated on GaP Waveguides

To demonstrate the summation operation by memlumors, sub-micrometer sized $CsPbBr_3$ crystals were integrated with GaP waveguides. For the signal collected from the edge of the waveguide, the synaptic weight is a property of the size of each crystal, its own PLQY and the waveguiding efficiency. The latter means that the same crystal located at different places of the waveguide should result in a different signal magnitude.

By diaphragming the excitation light input, it is possible to address independently different memlumors in the complex system (**Supplementary Fig. 15a**, **Supplementary Fig. 15b**) or illuminate them all at once by a broad excitation beam (**Supplementary Fig. 15c)**. In the latter case, the signal collected at the edge is a summation of the PL from all crystals guided to the waveguide edge.

References:

(1) Marunchenko, A.; Kondratiev, V.; Pushkarev, A.; Khubezhov, S.; Baranov, M.; Nasibulin, A.; Makarov, S. Mixed Ionic-Electronic Conduction Enables Halide-Perovskite Electroluminescent Photodetector. *Laser Photon. Rev.* **2023**, *17* (9). https://doi.org/10.1002/lpor.202300141.

(2) Trofimov, P.; Pushkarev, A. P.; Sinev, I. S.; Fedorov, V. V.; Bruyère, S.; Bolshakov, A.; Mukhin, I. S.; Makarov, S. V. Perovskite–Gallium Phosphide Platform for Reconfigurable Visible-Light Nanophotonic Chip. *ACS Nano* **2020**, *14* (7), 8126–8134. https://doi.org/10.1021/acsnano.0c01104.

(3) Taylor, A. D.; Sun, Q.; Goetz, K. P.; An, Q.; Schramm, T.; Hofstetter, Y.; Litterst, M.; Paulus, F.; Vaynzof, Y. A General Approach to High-Efficiency Perovskite Solar Cells by Any Antisolvent. *Nat. Commun.* **2021**, *12* (1), 1878. https://doi.org/10.1038/s41467-021-22049-8.

(4) Protesescu, L.; Yakunin, S.; Bodnarchuk, M. I.; Krieg, F.; Caputo, R.; Hendon, C. H.; Yang, R. X.; Walsh, A.; Kovalenko, M. V. Nanocrystals of Cesium Lead Halide Perovskites (CsPbX 3 , X = Cl, Br, and I): Novel Optoelectronic Materials Showing Bright Emission with Wide Color Gamut. *Nano Lett.* **2015**, *15* (6), 3692–3696. https://doi.org/10.1021/nl5048779.

(5) Kim, J.; Cho, S.; Dinic, F.; Choi, J.; Choi, C.; Jeong, S. M.; Lee, J.-S.; Voznyy, O.; Ko, M. J.; Kim, Y. Hydrophobic Stabilizer-Anchored Fully Inorganic Perovskite Quantum Dots Enhance Moisture Resistance and Photovoltaic Performance. *Nano Energy* **2020**, *75*, 104985. https://doi.org/10.1016/j.nanoen.2020.104985.

(6) Marunchenko, A.; Kumar, J.; Kiligaridis, A.; Tatarinov, D.; Pushkarev, A.; Vaynzof, Y.; Scheblykin, I. G. Memlumor: A Luminescent Memory Device for Energy-Efficient Photonic Neuromorphic Computing. *ACS Energy Lett.* **2024**, 2075–2082. https://doi.org/10.1021/acsenergylett.4c00691.

(7) Kiligaridis, A.; Frantsuzov, P. A.; Yangui, A.; Seth, S.; Li, J.; An, Q.; Vaynzof, Y.; Scheblykin, I. G. Are Shockley-Read-Hall and ABC Models Valid for Lead Halide Perovskites? *Nat. Commun.* **2021**, *12* (1), 3329. https://doi.org/10.1038/s41467-021-23275-w.

(8) Marunchenko, A.; Kumar, J.; Kiligaridis, A.; Tatarinov, D.; Pushkarev, A. P.; Vaynzof, Y.; Scheblykin, I. G. Memlumor: A Luminescent Memory Device for Energy Efficient Photonic Neuromorphic Computing. *http://arxiv.org/abs/2312.09170* **2024**.

(9) Ermolaev, G.; Pushkarev, A. P.; Zhizhchenko, A.; Kuchmizhak, A. A.; Iorsh, I.; Kruglov, I.; Mazitov, A.; Ishteev, A.; Konstantinova, K.; Saranin, D.; et al. Giant and Tunable Excitonic Optical Anisotropy in Single-Crystal Halide Perovskites. *Nano Lett.* **2023**, *23* (7), 2570–2577. https://doi.org/10.1021/acs.nanolett.2c04792.

(10) Liashenko, T. G.; Cherotchenko, E. D.; Pushkarev, A. P.; Pakštas, V.; Naujokaitis, A.; Khubezhov, S. A.; Polozkov, R. G.; Agapev, K. B.; Zakhidov, A. A.; Shelykh, I. A.; et al. Electronic Structure of CsPbBr 3−x Cl x Perovskites: Synthesis, Experimental Characterization, and DFT Simulations. *Phys. Chem. Chem. Phys.* **2019**, *21* (35), 18930–18938. https://doi.org/10.1039/C9CP03656C.

(11) Lakowicz, J. R. *Principles of Fluorescence Spectroscopy*; Springer US: Boston, MA, 2006. https://doi.org/10.1007/978-0-387-46312-4.

(12) Rao, S. M.; Kiligaridis, A.; Yangui, A.; An, Q.; Vaynzof, Y.; Scheblykin, I. G. Photoluminescence Mapping over Laser Pulse Fluence and Repetition Rate as a Fingerprint of Charge and Defect Dynamics in Perovskites. *Adv. Opt. Mater.* **2023**, 2300996. https://doi.org/10.1002/adom.202300996.

(13) Abakumov, V. N.; Perel, V. I.; Yassievich, I. N. *Nonradiative Recombination in Semiconductors*; North-Holland, Amsterdam, 1991.
